# Supplementary material for: RanBP2-dependent annulate lamellae drive nuclear pore assembly and nuclear expansion
Source: Nat Commun. 2026 Mar 25;17:4400. doi: 10.1038/s41467-026-71101-y (PMC13180977; doi:10.1038/s41467-026-71101-y)
Supplement: Supplementary file 2 — Description of Additional Supplementary Files [file 41467_2026_71101_MOESM2_ESM.pdf]

Description of Additional Supplementary Files

File name: Supplementary Video 1
Description: AL foci cluster following MT depolymerization. 2xZFN-mEGFP-Nup107 HeLa cells treated with nocodazole were analyzed by live spinning-disk confocal microscopy. Z-stacks spanning a 10 µm range with a 0.5 µm step size were acquired every 10 min. Maximum-intensity projection images are shown at 5 frames per second. A representative cell is shown, and selected frames from this video are displayed in Extended Data Fig. 5c.

File name: Supplementary Video 2
Description: AL foci merge with the NE throughout interphase. 2xZFN-mEGFP-Nup107 HeLa cells were synchronized by double thymidine block, released, and analyzed by live spinning-disk confocal microscopy. Time 0 indicates mitotic exit and entry into a new cell cycle. Z-stacks spanning a 10 µm range with a 0.5 µm step size were acquired every 5 min. Maximum-intensity projection images are shown at 5 frames per second.

File name: Supplementary Video 3
Description: AL foci are highly dynamic under physiological conditions. 2xZFN-mEGFP-Nup107 HeLa cells were analyzed by live spinning-disk confocal microscopy. Z-stacks spanning a 5 µm range with a 1 µm step size were acquired every 1 s. Maximum-intensity projection images are shown at 5 frames per second. A representative cell is shown, and selected frames from this video are displayed in Fig. 2e.

File name: Supplementary Video 4
Description: Smaller AL foci fuse more rapidly with the NE than larger AL. 2xZFN-mEGFP-Nup107 HeLa cells were analyzed by live spinning-disk confocal microscopy. Z-stacks spanning a 5 µm range with a 1 µm step size were acquired every 3 s. Maximum-intensity projection images are shown at 5 frames per second. Three representative cells are shown, and selected frames from this video are displayed in Fig. 2e.

File name: Supplementary Video 5
Description: AL can undergo multiple cycles of detachment and re-attachment before fusing with the NE. 2xZFN-mEGFP-Nup107 HeLa cells were analyzed by live spinning-disk confocal microscopy. Z-stacks spanning a 5 µm range with a 1 µm step size were acquired every 3 s. Maximum-intensity projection images are shown at 5 frames per second. A representative cell is shown, and selected frames from this video are displayed in Fig. 2e.

File name: Supplementary Video 6
Description: NE-NPCs do not diffuse from the NE into the cytoplasm. mEOS2-Nup133 HeLa cells were analyzed by live spinning-disk confocal microscopy. Nup133 at the nuclear envelope was photoconverted from green fluorescence (left channel) to red fluorescence (right channel) by blue light illumination for 30 s. Z-stacks spanning a 10 µm range with a 1 µm step size were acquired. A 30 s pre-acquisition sequence was recorded at 10 s intervals in both green and red channels. Following photoconversion, two post-acquisition sequences were performed: a short sequence of 30 s acquired every 10 s to monitor immediate effects, and a long-term sequence acquired every 15 min for 12 h to assess longer-term behavior. Maximum-intensity projection images are shown at 5 frames per second.

File name: Supplementary Video 7
Description: AL-NPCs accumulate at the NE over time. mEOS2-Nup133 HeLa cells were analyzed by live spinning-disk confocal microscopy. Cytoplasmic Nup133 was photoconverted from green fluorescence (left channel) to red fluorescence (right channel) by blue light illumination for 30 s. Z-stacks spanning a 10 µm range with a 1 µm step size

were acquired. A 30 s pre-acquisition sequence was recorded at 10 s intervals in both green and red channels. Following photoconversion, two post-acquisition sequences were performed: a short sequence of 30 s acquired every 10 s to monitor immediate effects, and a long-term sequence acquired every 15 min for 12 h to assess accumulation over time. Maximum-intensity projection images are shown at 5 frames per second.

File name: Supplementary Video 8
Description: AL foci move along the ER to merge with the NE. 2xZFN-mEGFP-Nup107 HeLa cells expressing the ER marker mScarlet-ER were analyzed by live spinning-disk confocal microscopy. Time 0 indicates the start of imaging. Frames were acquired every 20 s without Z-stacks. Images are shown at 5 frames per second. A representative cell is shown in Fig. 3d, and zoomed regions of selected frames from this video are displayed.

File name: Supplementary Video 9
Description: Microtubule depolymerization inhibits AL foci integration into the NE and promotes their clustering. 2xZFN-mEGFP-Nup107 HeLa cells expressing the ER marker mScarlet-ER were analyzed by live spinning-disk confocal microscopy. Time 0 indicates the start of imaging. Frames were acquired every 120 s without Z-stacks. Images are shown at 5 frames per second. A representative cell is shown in Fig. 3d, and zoomed regions of selected frames from this video are displayed.

File name: Supplementary Video 10
Description: AL foci associate with microtubules. 2xZFN-mEGFP-Nup107 HeLa cells were analyzed by live spinning-disk confocal microscopy. Microtubules were labelled with SiR-tubulin. Frames were acquired every 2 s without Z-stacks. Images are shown at 5 frames per second.
