# Supplementary material for: RanBP2-dependent annulate lamellae drive nuclear pore assembly and nuclear expansion
Source: Nat Commun. 2026 Mar 25;17:4400. doi: 10.1038/s41467-026-71101-y (PMC13180977; doi:10.1038/s41467-026-71101-y)
Supplement: Supplementary file 13 — Reporting Summary [file 41467_2026_71101_MOESM13_ESM.pdf]

nature portfolio

Corresponding author(s): Izabela Sumara

Last updated by author(s): Jan 13, 2026

Reporting Summary

Nature Portfolio wishes to improve the reproducibility of the work that we publish. This form provides structure for consistency and transparency in reporting. For further information on Nature Portfolio policies, see our Editorial Policies and the Editorial Policy Checklist.

Statistics

For all statistical analyses, confirm that the following items are present in the figure legend, table legend, main text, or Methods section.

n/a	Confirmed	
☐	☒	The exact sample size (*n*) for each experimental group/condition, given as a discrete number and unit of measurement
☐	☒	A statement on whether measurements were taken from distinct samples or whether the same sample was measured repeatedly
☐	☒	The statistical test(s) used AND whether they are one- or two-sided<br>*Only common tests should be described solely by name; describe more complex techniques in the Methods section.*
☒	☐	A description of all covariates tested
☐	☒	A description of any assumptions or corrections, such as tests of normality and adjustment for multiple comparisons
☐	☒	A full description of the statistical parameters including central tendency (e.g. means) or other basic estimates (e.g. regression coefficient) AND variation (e.g. standard deviation) or associated estimates of uncertainty (e.g. confidence intervals)
☐	☒	For null hypothesis testing, the test statistic (e.g. *F*, *t*, *r*) with confidence intervals, effect sizes, degrees of freedom and *P* value noted<br>*Give P values as exact values whenever suitable.*
☒	☐	For Bayesian analysis, information on the choice of priors and Markov chain Monte Carlo settings
☒	☐	For hierarchical and complex designs, identification of the appropriate level for tests and full reporting of outcomes
☒	☐	Estimates of effect sizes (e.g. Cohen's *d*, Pearson's *r*), indicating how they were calculated

Our web collection on statistics for biologists contains articles on many of the points above.

Software and code

Policy information about availability of computer code

Data collection	No custom software was generated for data collection
Data analysis	Only commercial or publicly available software was used for data analysis. Image quantification analysis was performed using ImageJ (1.46r)or CellProfiler (4.2.6). Statistical analysis was performed using GraphPad Prism (9.0.0). Single-molecule localization microscopy data was processed using the Leica LAS X and SharpVisu (https://github.com/andronovl/SharpViSu) while omitting the spectral demixing steps in the SplitVisu plugin. TEM tomography was performed on the areas of interest using the software package SerialEM8. Tomograms were reconstructed with IMOD9. Correlation between LM and EM images was done with the plugin ec-CLEM10 of the software platform Icy. MS raw files were processed using Spectronaut 18 (Biognosys, Switzerland). Multivariate statistics on protein measurements were performed using Qlucore Omics Explorer 3.9 (Qlucore AB, Lund, SWEDEN).

For manuscripts utilizing custom algorithms or software that are central to the research but not yet described in published literature, software must be made available to editors and reviewers. We strongly encourage code deposition in a community repository (e.g. GitHub). See the Nature Portfolio guidelines for submitting code & software for further information.

Data

Policy information about availability of data

All manuscripts must include a data availability statement. This statement should provide the following information, where applicable:

- Accession codes, unique identifiers, or web links for publicly available datasets
- A description of any restrictions on data availability
- For clinical datasets or third party data, please ensure that the statement adheres to our policy

Provide your data availability statement here.

Research involving human participants, their data, or biological material

Policy information about studies with human participants or human data. See also policy information about sex, gender (identity/presentation), and sexual orientation and race, ethnicity and racism.

Reporting on sex and gender	N/A
Reporting on race, ethnicity, or other socially relevant groupings	N/A
Population characteristics	N/A
Recruitment	N/A
Ethics oversight	N/A

Note that full information on the approval of the study protocol must also be provided in the manuscript.

Field-specific reporting

Please select the one below that is the best fit for your research. If you are not sure, read the appropriate sections before making your selection.

☒ Life sciences ☐ Behavioural & social sciences ☐ Ecological, evolutionary & environmental sciences

For a reference copy of the document with all sections, see nature.com/documents/nr-reporting-summary-flat.pdf

Life sciences study design

All studies must disclose on these points even when the disclosure is negative.

Sample size	Sample size (or number of repeats) was chosen based on what is common in the field, and what was practical to do. A minimum of three independent replicates was performed for each experiment in order to add statistical analysis, when required, as stated in the Methods section of the manuscript.
Data exclusions	In general, no data were excluded unless there was a valid reason to do so, e.g. experiments with failed positive controls indicating technical problems, or other technical issues.
Replication	Most biochemical experiments were performed at least three times, as defined in figure legends. Experiments that could not be reproduced are not presented in this study.
Randomization	N/A
Blinding	N/A

Reporting for specific materials, systems and methods

We require information from authors about some types of materials, experimental systems and methods used in many studies. Here, indicate whether each material, system or method listed is relevant to your study. If you are not sure if a list item applies to your research, read the appropriate section before selecting a response.

Materials & experimental systems

n/a	Involved in the study	
☐	☒	Antibodies
☐	☒	Eukaryotic cell lines
☒	☐	Palaeontology and archaeology
☒	☐	Animals and other organisms
☒	☐	Clinical data
☒	☐	Dual use research of concern
☒	☐	Plants

Methods

n/a	Involved in the study	
☒	☐	ChIP-seq
☒	☐	Flow cytometry
☒	☐	MRI-based neuroimaging

Antibodies

Antibodies used

rabbit monoclonal anti-Nup133 (Abcam, ab155990), mouse monoclonal anti-Nup133 (E-6) (Santa Cruz Biotechnology, sc-376763), mouse monoclonal anti-Nucleoporin p62 (BD Biosciences, 610497), rabbit polyclonal anti-RanBP2 (Abcam, ab64276), rabbit polyclonal anti-Nup214 (Abcam, ab70497), rabbit polyclonal anti-Nup85 (Bethyl, A303-977A), mouse monoclonal anti-Pericentrin 1 (D-4) (Nup85) (Santa Cruz Biotechnology, sc-376111), rabbit monoclonal anti-SEC13 (R&D systems, MAB9055), rabbit polyclonal anti-ELYS (Bethyl, A300-166A), mouse monoclonal Nup205 (H-1) (Santa Cruz Biotechnology, sc-377047), mouse monoclonal anti-Nup93 (E-8) (Santa Cruz Biotechnology, sc-374399), rabbit monoclonal anti-Nup98 (C39A3) (Cell Signaling Technology, 2598), rabbit polyclonal anti-Nup96 (Bethyl, A301-784A), rabbit polyclonal anti-POM121 (GeneTex, GTX102128), rabbit polyclonal anti-Nup153 (Abcam, ab84872), rabbit polyclonal anti-CRM1/Exportin 1 (Novus, NB100-79802), mouse monoclonal anti-NTF97/Importin beta (Abcam, ab2811), mouse monoclonal anti-Ran (BD Biosciences, 610340), rabbit monoclonal anti-SUN1 (Abcam, ab124770), rabbit monoclonal anti-SUN2 (Abcam, ab124916), rabbit polyclonal anti-Lamin A (C-terminal) (Sigma, L1293), rabbit polyclonal anti-Lamin B1 (Abcam, ab16048), rabbit monoclonal anti-Nesprin1 (Abcam, ab192234), rabbit polyclonal anti-Emerin (Abcam, ab40688), rabbit polyclonal anti-LAP2 (Proteintech, 14651-1-AP), rabbit polyclonal anti-Phospho-Rb (Ser807/811) (Cell Signaling Technology, 9308), mouse monoclonal anti-Cyclin B1 (G-11) (Santa Cruz Biotechnology, sc-166757), rabbit polyclonal anti-Cyclin B1 (GeneTex, GTX100911), rat monoclonal anti-HA (Roche, 11867423001), rabbit polyclonal anti-GAPDH (Sigma, G9545), mouse monoclonal anti-β-Actin (Sigma, A2228), mouse monoclonal anti-mCherry (4B3) (Thermo Scientific, MA5-32977), rabbit polyclonal anti-UBAP2L (Abcam, ab138309), mouse monoclonal anti-FXR1 (Millipore, 03-176), rabbit polyclonal anti-α-Tubulin (Abcam, ab18251), mouse monoclonal anti-α-Tubulin (Sigma, T9026), mouse monoclonal anti-Nuclear Pore Complex Proteins (mAb414) (Abcam, ab24609), rabbit polyclonal anti-LC3B (Novus biological, NB100-2220SS), rabbit polyclonal anti-LC3A (Novus biological, NB100-2331), rabbit polyclonal anti-p62 (GeneTex, GTX100685), rat monoclonal anti-GFP (3H9) (ChromoTek, 3h9-100), rabbit polyclonal anti-GFP (Abcam, ab290), rabbit polyclonal anti-Aurora B (Abcam, ab2254), rabbit polyclonal anti-cyclin A (H-432) (Santa Cruz Biotechnology, sc-751), mouse monoclonal anti-PDI (Abcam, ab2792), mouse monoclonal anti-Climp63 (Enzo, ALX-804-604), rabbit polyclonal anti-CKAP4 (Proteintech, 16686-1-AP), rat monoclonal α-tubulin-conjugated to Alexa Fluor® 647 (Abcam, ab195884), rabbit polyclonal anti-RTN4/NOGO (Proteintech, 10950-1-AP), rabbit polyclonal anti-RRBP1 (Proteintech, 22015-1-AP), rabbit polyclonal anti- KTN1 (Proteintech, 19841-1-AP), rabbit polyclonal anti-ATL1(OriGene, ta332595), rabbit polyclonal anti-ATL3 (Proteintech, 16921-1-AP), rabbit polyclonal anti-LNPK (Atlas Antibodies, HPA014205).

Validation

Antibody validation information can be found at the manufacturer's website according to the provided item number.

Eukaryotic cell lines

Policy information about cell lines and Sex and Gender in Research

Cell line source(s)

HeLa Kyoto (human cervix carcinoma) cells and U2OS (human bone osteosarcoma) cells were purchased from ATCC. HeLa Kyoto GFP-Nup107 derived stable cell were purchased from CSL cell bank. AID-NUP358 DLD-1 (colorectal adenocarcinoma epithelial cells) cells and AID-NUP358 HTC116 (human colon cancer cells) cells were generous gifts from Mary Dasso (National Institutes of Health). hTERT-RPE1 (Human retinal pigment epithelial-1) cells was a generous gift from Juliette Godin (IGBMC). FB789 (Human Fibroblast) cells was a generous gift from PROIETTI Luca (IGBMC). MRC5 (Human Fibroblast) cells was a generous gift from SEROZ Thierry (IGBMC). Control human fibroblasts and FXS patient-derived fibroblasts were previously reported (DOI: 10.1093/hmg/ddy099).Human induced pluripotent stem cells (hiPSCs, GM8330-8) as a generous gift from M. E. Talkowski)

Authentication

HeLa Kyoto and U2OS were validated at ATCC or CSL cell bank.
Modified cell lines were confirmed by immunoblotting and/or genomic sequencing for the protein/gene of interest.
hTERT-RPE1, FB789, MRC5 and hiPSCs were confirmed by the cell culture platform of the IGBMC (Strasbourg).
Other cells were verified at the source: AID-NUP358 DLD-1 and AID-NUP358 HTC116 (DOI: 10.1126/science.abm9129); Control human fibroblasts and FXS patient-derived fibroblasts (DOI: 10.1093/hmg/ddy099).

Mycoplasma contamination

We routinely test for mycoplasm for all cell lines. Al the experiments performed here were using mycoplasm-free cell lines

Commonly misidentified lines
(See ICLAC register)

N/A

Plants

Seed stocks	N/A
Novel plant genotypes	N/A
Authentication	N/A
