# Supplementary material for: RanBP2-dependent annulate lamellae drive nuclear pore assembly and nuclear expansion
Source: Nat Commun. 2026 Mar 25;17:4400. doi: 10.1038/s41467-026-71101-y (PMC13180977; doi:10.1038/s41467-026-71101-y)
Supplement: Supplementary file 1 — Supplementary Information [file 41467_2026_71101_MOESM1_ESM.pdf]

Supplementary Information for

RanBP2-dependent annulate lamellae drive nuclear pore assembly and nuclear expansion

Junyan Lin, Arantxa Agote-Aran, Yongrong Liao, Mehdi Cloarec, Leonid Andronov, Rafael L. Schoch, Paolo Ronchi, Victor Cochard, Rui Zhu, Erwan Grandgirard, Xiaotian Liu, Marianne Victoria Lemée, Charlotte Kleiss, Christelle Golzio, Marc Ruff, Guillaume Chevreux, Yannick Schwab, Bruno P. Klaholz and Izabela Sumara

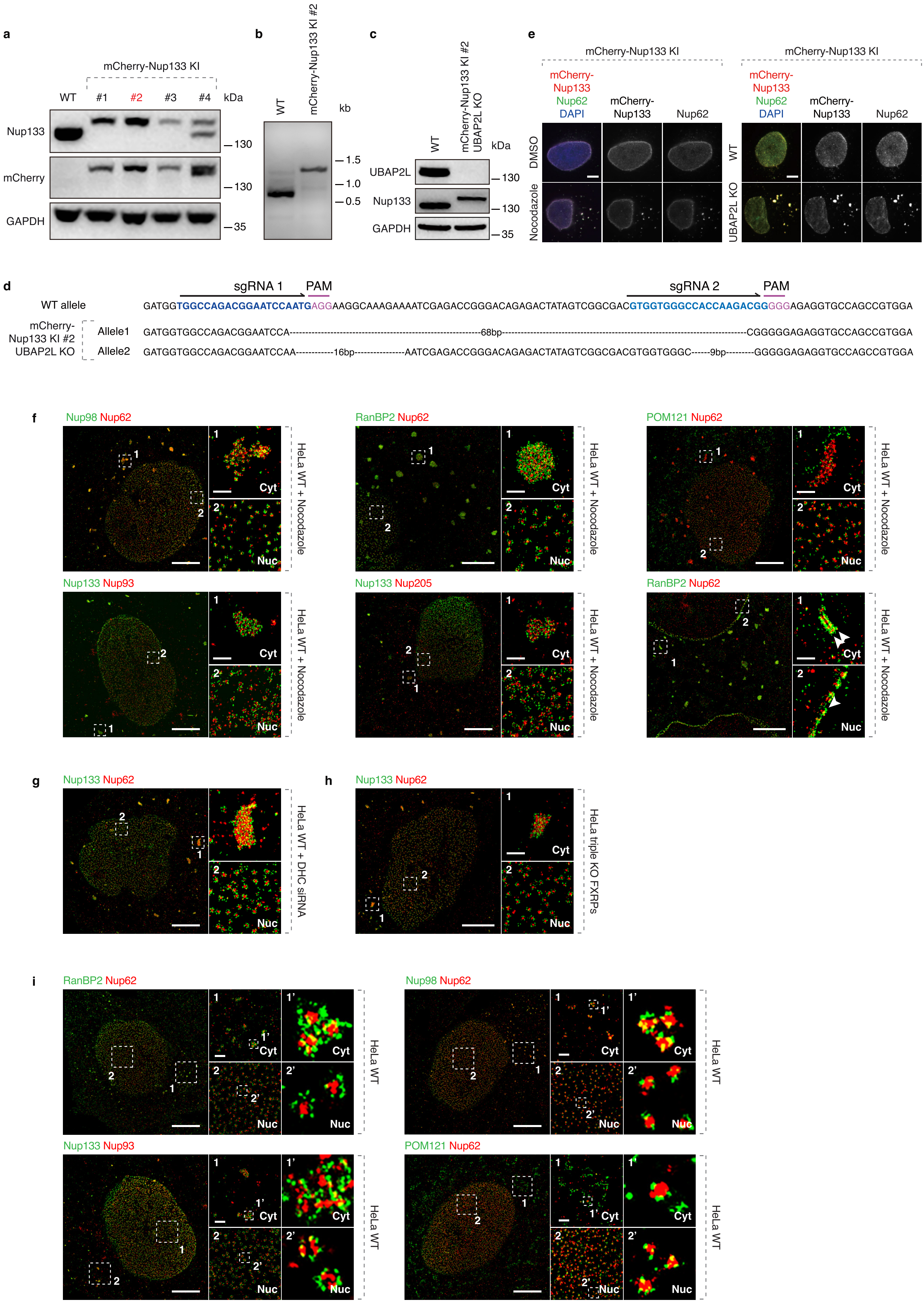

a
mCherry-Nup133 KI
WT
#1
#2
#3
#4
kDa
Nup133
mCherry
GAPDH
130
130
35
b
WT
mCherry-Nup133 KI #2
kb
1.5
1.0
0.5
c
WT
mCherry-Nup133 KI #2 UBAP2L KO
kDa
UBAP2L
Nup133
GAPDH
130
130
35
e
mCherry-Nup133 KI
mCherry-Nup133 Nup62 DAPI
mCherry-Nup133
Nup62
DMSO
Nocodazole
WT
UBAP2L KO
d
sgRNA 1
PAM
sgRNA 2
PAM
WT allele
GATGGTGGCCAGACGGAATCCAATGAGGAAGGCAAAGAAAATCGAGACCGGGACAGAGACTATAGTCGGCGACGTGGTGGGCCACCAAGACGGGGGAGAGGTGCCAGCCGTGGA
mCherry-Nup133 KI #2 UBAP2L KO
Allele1
GATGGTGGCCAGACGGAATCCA---68bp---CGGGGGAGAGGTGCCAGCCGTGGA
Allele2
GATGGTGGCCAGACGGAATCCAA---16bp---AATCGAGACCGGGACAGAGACTATAGTCGGCGACGTGGTGGGC---9bp---GGGGGAGAGGTGCCAGCCGTGGA
f
Nup98 Nup62
RanBP2 Nup62
POM121 Nup62
Nup133 Nup93
Nup133 Nup205
RanBP2 Nup62
Cyt
Nuc
HeLa WT + Nocodazole
g
Nup133 Nup62
HeLa WT + DHC siRNA
h
Nup133 Nup62
HeLa triple KO FXRPs
i
RanBP2 Nup62
Nup98 Nup62
Nup133 Nup93
POM121 Nup62
HeLa WT

Supplementary Fig. 1. **Single molecule localization microscopy identifies AL in WT and challenged HeLa cells. a, b** Validation of CRISPR-Cas9-mediated mCherry-Nup133 knock-in (KI) HeLa cell clones by western blotting (a) and PCR (b). **c, d** Validation of CRISPR-Cas9-mediated UBAP2L knockout (KO) mCherry-Nup133 KI HeLa cell clones by western blotting (c) and Sanger sequencing (d). **e** Representative images of mCherry-Nup133 KI HeLa cells treated with DMSO or nocodazole (10 µM) and of UBAP2L KO mCherry-Nup133 KI HeLa cells. Cells were co-labelled with DAPI (blue) and anti-Nup62 (green) antibodies. Scale bars, 5 µm. **f** Representative splitSMLM images showing NPCs at the nuclear (Nuc) surface and AL-NPCs in the cytoplasm (Cyt) of nocodazole-treated HeLa cells (10 µM, 90 min). Nups from different NPC subcomplexes analyzed are indicated above each panel. Magnified boxed regions are shown in the corresponding numbered panels. Scale bars, 3 µm (entire nuclei) and 0.3 µm (zoom). **g, h** Representative splitSMLM images showing NPCs at the nuclear (Nuc) surface and AL-NPCs in the cytoplasm (Cyt) of HeLa cells treated with dynein heavy chain siRNAs for 48 h and of FXRP triple-knockout HeLa cells. Nup133 labels the cytoplasmic and nuclear rings of NPCs, and Nup62 marks the central channel. Magnified boxed regions are shown in the corresponding numbered panels. Scale bars, 3 µm (entire nuclei) and 0.3 µm (zoom). **i** Representative splitSMLM images showing NPCs at the nuclear (Nuc) surface and AL-NPCs in the cytoplasm (Cyt) of WT HeLa cells. Nups from different NPC subcomplexes analyzed are indicated above each panel. Magnified boxed regions are shown in the corresponding numbered panels. Scale bars, 3 µm (entire nuclei) and 0.3 µm (zoom).

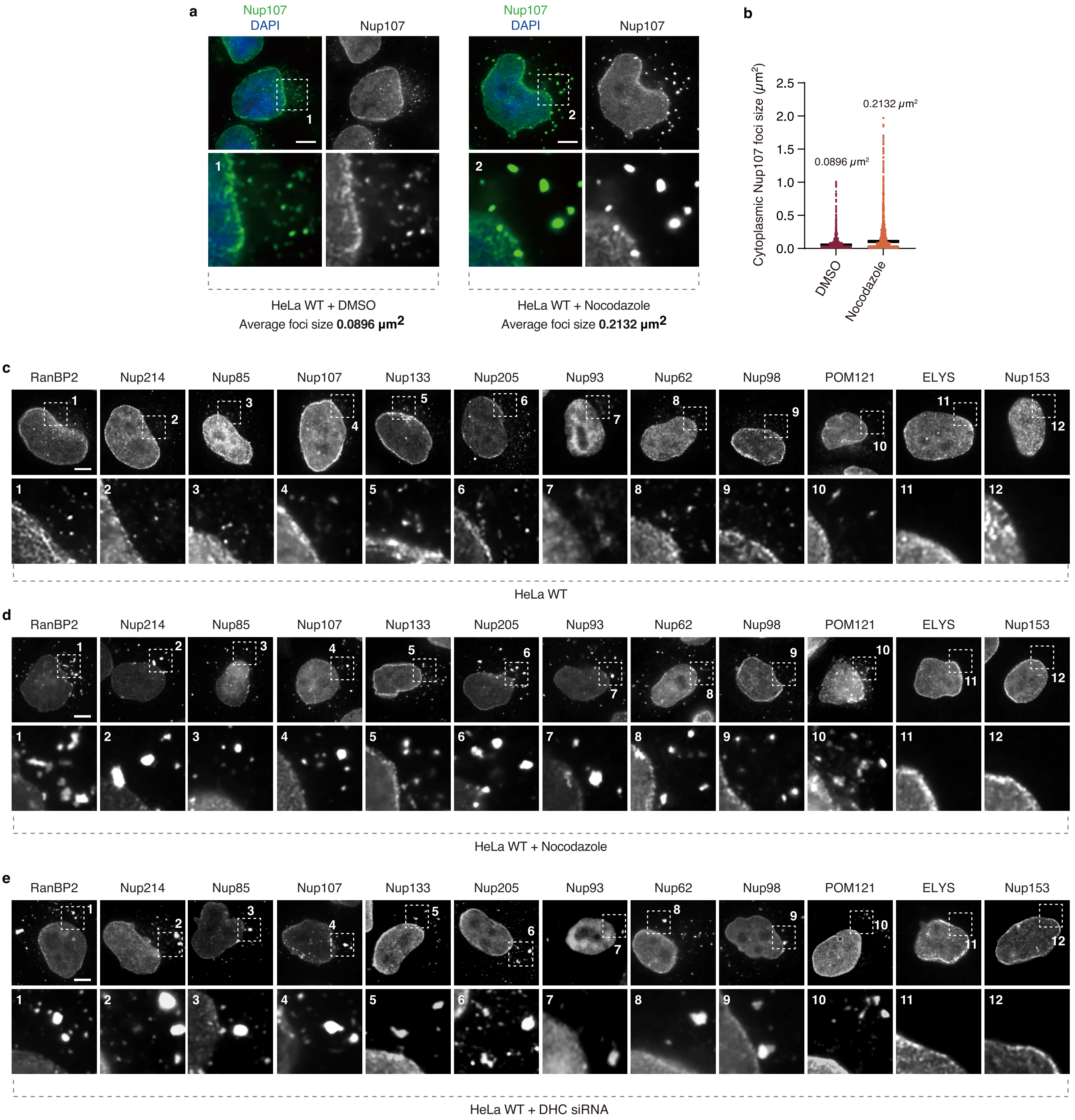

f

		NE-NPCs	*AL-NPCs WT*	*AL-NPCs Nocodazole*	*AL-NPCs DHC siRNA*	*AL-NPCs FXR1 siRNA**	*AL-NPCs UBAP2L KO**
Cytoplasmic filaments	RanBP2	Y	Y	Y	Y	Y	Y
	Nup214	Y	Y	Y	Y	Y	-
Outer rings	Nup85	Y	Y	Y	Y	Y	Y
	Nup107	Y	Y	Y	Y	Y	Y
	Nup133	Y	Y	Y	Y	Y	Y
	ELYS	**Y**	**N**	**N**	**N**	**N**	**N**
Inner rings	Nup205	Y	Y	Y	Y	-	Y
	Nup93	Y	Y	Y	Y	-	Y
Central channel	Nup62	Y	Y	Y	Y	Y	Y
Linker Nups	Nup98	Y	Y	Y	Y	Y	Y
Transmembrane Nups	POM121	Y	Y	Y	Y	Y	Y
Nuclear basket	Nup153	**Y**	**N**	**N**	**N**	**N**	**N**

Supplementary Fig. 2. **Systematic analysis of the size and composition of AL foci. a, b** Representative images of 2xZFN-mEGFP-Nup107 HeLa cells treated with nocodazole (10 μM) or DMSO for 90 min (a). Cells were co-labelled with DAPI (blue). Magnified boxed regions are shown in the corresponding numbered panels. The size of cytoplasmic Nup107-positive AL foci was quantified (b); at least 2000 foci per condition were analyzed (N = 3). Scale bars, 5 μm. Source data are provided as a Source Data file. **c** Representative images of WT HeLa cells labelled with Nups from different NPC subcomplexes. The analyzed Nups are indicated above each panel. Magnified boxed regions are shown in the corresponding numbered panels. Scale bar, 5 μm. **d** Representative images of nocodazole-treated HeLa cells (10 μM, 90 min) labelled with Nups from different NPC subcomplexes. The analyzed Nups are indicated above each panel. Magnified boxed regions are shown in the corresponding numbered panels. Scale bar, 5 μm. **e** Representative images of HeLa cells treated with dynein heavy chain siRNAs for 48 h and labelled with Nups from different NPC subcomplexes. The analyzed Nups are indicated above each panel. Magnified boxed regions are shown in the corresponding numbered panels. Scale bar, 5 μm. **f** Summary of the composition of NE-NPCs and AL-NPCs under different conditions. Different colors represent specific NPC subcomplexes. Y indicates presence and N indicates absence of the indicated nucleoporins. A dash indicates no available data. An asterisk indicates previously published results[25,27,28].

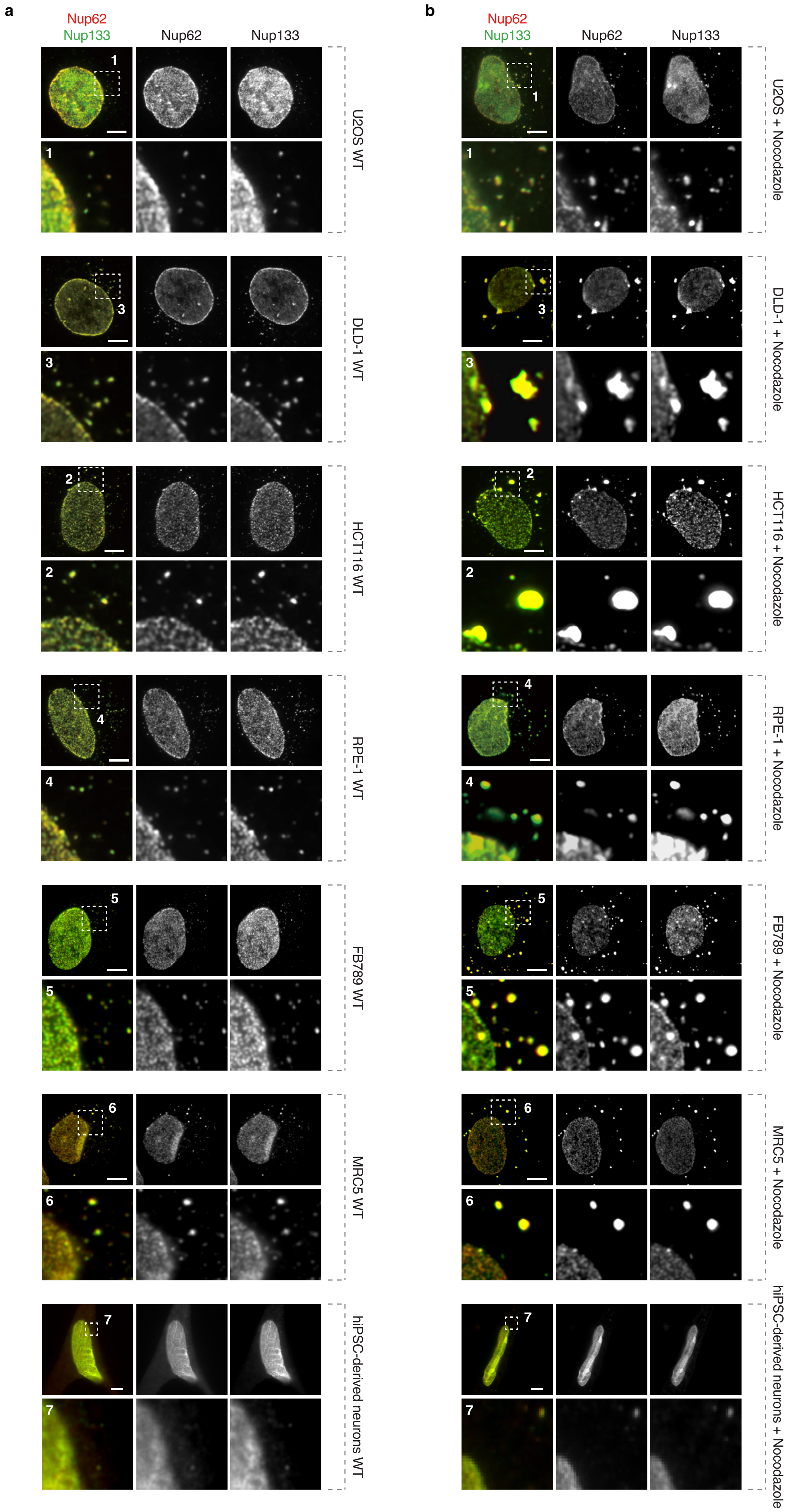

a
Nup62
Nup133
Nup62
Nup133
U2OS WT
DLD-1 WT
HCT116 WT
RPE-1 WT
FB789 WT
MRC5 WT
hiPSC-derived neurons WT
1
2
3
4
5
6
7
b
Nup62
Nup133
Nup62
Nup133
U2OS + Nocodazole
DLD-1 + Nocodazole
HCT116 + Nocodazole
RPE-1 + Nocodazole
FB789 + Nocodazole
MRC5 + Nocodazole
hiPSC-derived neurons + Nocodazole

Supplementary Fig. 3. **AL exist in diverse somatic mammalian cells. a** Representative images of WT U2OS, DLD-1, HCT116, RPE-1, FB789, MRC5, and hiPSC-derived neurons showing AL foci. Cells were co-labelled with anti-Nup62 (red) and anti-Nup133 (green) antibodies to visualize AL-NPCs. Scale bars, 5 µm. **b** Representative images of U2OS, DLD-1, HCT116, RPE-1, FB789, MRC5, and hiPSC-derived neurons treated with nocodazole (10 µM, 90 min). Cells were co-labelled with anti-Nup62 (red) and anti-Nup133 (green) antibodies to visualize AL-NPCs. Scale bars, 5 µm.

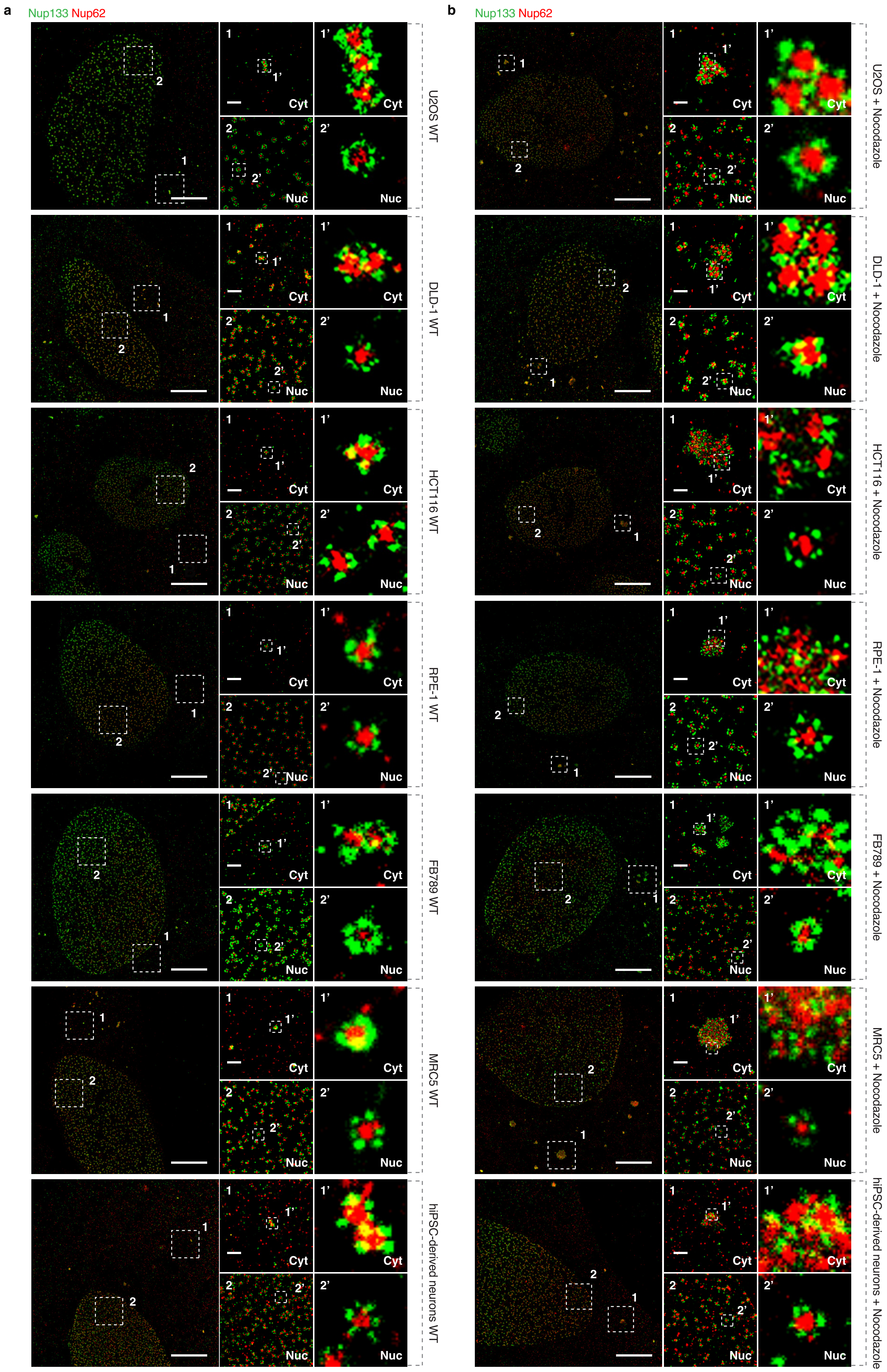

a
Nup133 Nup62
b
Nup133 Nup62
1
2
1'
2'
Cyt
Nuc
U2OS WT
DLD-1 WT
HCT116 WT
RPE-1 WT
FB789 WT
MRC5 WT
hiPSC-derived neurons WT
U2OS + Nocodazole
DLD-1 + Nocodazole
HCT116 + Nocodazole
RPE-1 + Nocodazole
FB789 + Nocodazole
MRC5 + Nocodazole
hiPSC-derived neurons + Nocodazole

Supplementary Fig. 4. **AL exist in diverse somatic mammalian cells. a** Representative splitSMLM images showing NPCs at the nuclear (Nuc) surface and AL-NPCs in the cytoplasm (Cyt) of WT U2OS, DLD-1, HCT116, RPE-1, FB789, MRC5, and hiPSC-derived neurons. Analyzed Nups from different NPC subcomplexes are indicated above each panel. Magnified boxed regions are shown in the corresponding numbered panels. Scale bars, 3 µm (entire nuclei) and 0.3 µm (zoom). **b** Representative splitSMLM images showing NPCs at the nuclear (Nuc) surface and AL-NPCs in the cytoplasm (Cyt) of nocodazole-treated U2OS, DLD-1, HCT116, RPE-1, FB789, MRC5, and hiPSC-derived neurons (10 µM, 90 min). Analyzed Nups from different NPC subcomplexes are indicated above each panel. Magnified boxed regions are shown in the corresponding numbered panels. Scale bars, 3 µm (entire nuclei) and 0.3 µm (zoom).

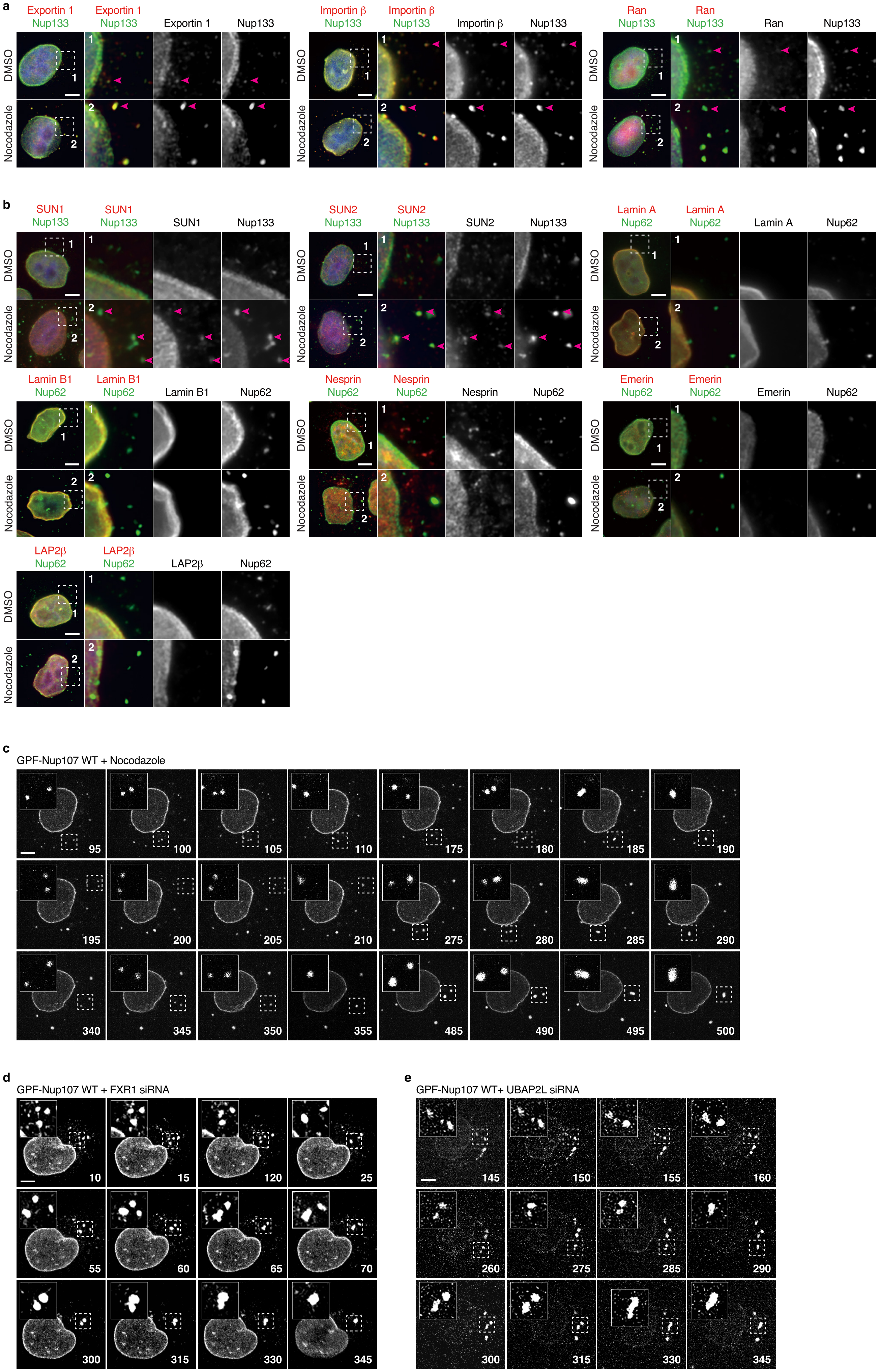
a
Exportin 1 Nup133
Exportin 1 Nup133
Exportin 1
Nup133
Importin β Nup133
Importin β Nup133
Importin β
Nup133
Ran Nup133
Ran Nup133
Ran
Nup133
DMSO
Nocodazole
b
SUN1 Nup133
SUN1 Nup133
SUN1
Nup133
SUN2 Nup133
SUN2 Nup133
SUN2
Nup133
Lamin A Nup62
Lamin A Nup62
Lamin A
Nup62
Lamin B1 Nup62
Lamin B1 Nup62
Lamin B1
Nup62
Nesprin Nup62
Nesprin Nup62
Nesprin
Nup62
Emerin Nup62
Emerin Nup62
Emerin
Nup62
LAP2β Nup62
LAP2β Nup62
LAP2β
Nup62
c
GPF-Nup107 WT + Nocodazole
95 100 105 110 175 180 185 190
195 200 205 210 275 280 285 290
340 345 350 355 485 490 495 500
d
GPF-Nup107 WT + FXR1 siRNA
10 15 120 25
55 60 65 70
300 315 330 345
e
GPF-Nup107 WT+ UBAP2L siRNA
145 150 155 160
260 275 285 290
300 315 330 345

Supplementary Fig. 5. **Analysis of AL composition and AL-NPC clustering. a** Representative images showing co-localization of nucleocytoplasmic transport factors (Exportin-1, Importin β, and Ran) with AL-NPC foci in HeLa cells treated with nocodazole (10 μM) or DMSO for 90 min. Cells were co-labelled with anti-transport factor (Exportin-1, Importin β, and Ran) antibodies (red) and anti-Nup133 (green). Magnified boxed regions are shown in the corresponding numbered panels. Magenta arrows indicate co-localized AL and transport factor foci. Scale bars, 5 μm. **b** Representative images showing co-localization of NE-associated proteins (SUN1, SUN2, Lamin A, Lamin B1, Nesprin, Emerin, and Lap2) with AL-NPC foci in HeLa cells treated with nocodazole (10 μM) or DMSO for 90 min. Cells were co-labelled with anti-NE-related protein antibodies (red) and anti-Nup62 (green). Magnified boxed regions are shown in the corresponding numbered panels. Magenta arrows indicate co-localized AL and NE factor foci. Scale bars, 5 μm. **c** 2xZFN-mEGFP-Nup107 HeLa cells treated with nocodazole were analyzed by live spinning-disk confocal microscopy. Selected representative frames are shown, with time indicated in minutes relative to nocodazole addition (time 0). Magnified boxed regions are shown in the upper left corner. Scale bar, 5 μm. **d, e** 2xZFN-mEGFP-Nup107 HeLa cells treated with FXR1 siRNA (d) or UBAP2L siRNA (e) were analyzed by live spinning-disk confocal microscopy. Selected representative frames are shown, with time indicated in minutes. Time 0 corresponds to 12 h after release from a double thymidine block. Magnified boxed regions are shown in the upper left corner. Scale bars, 5 μm.

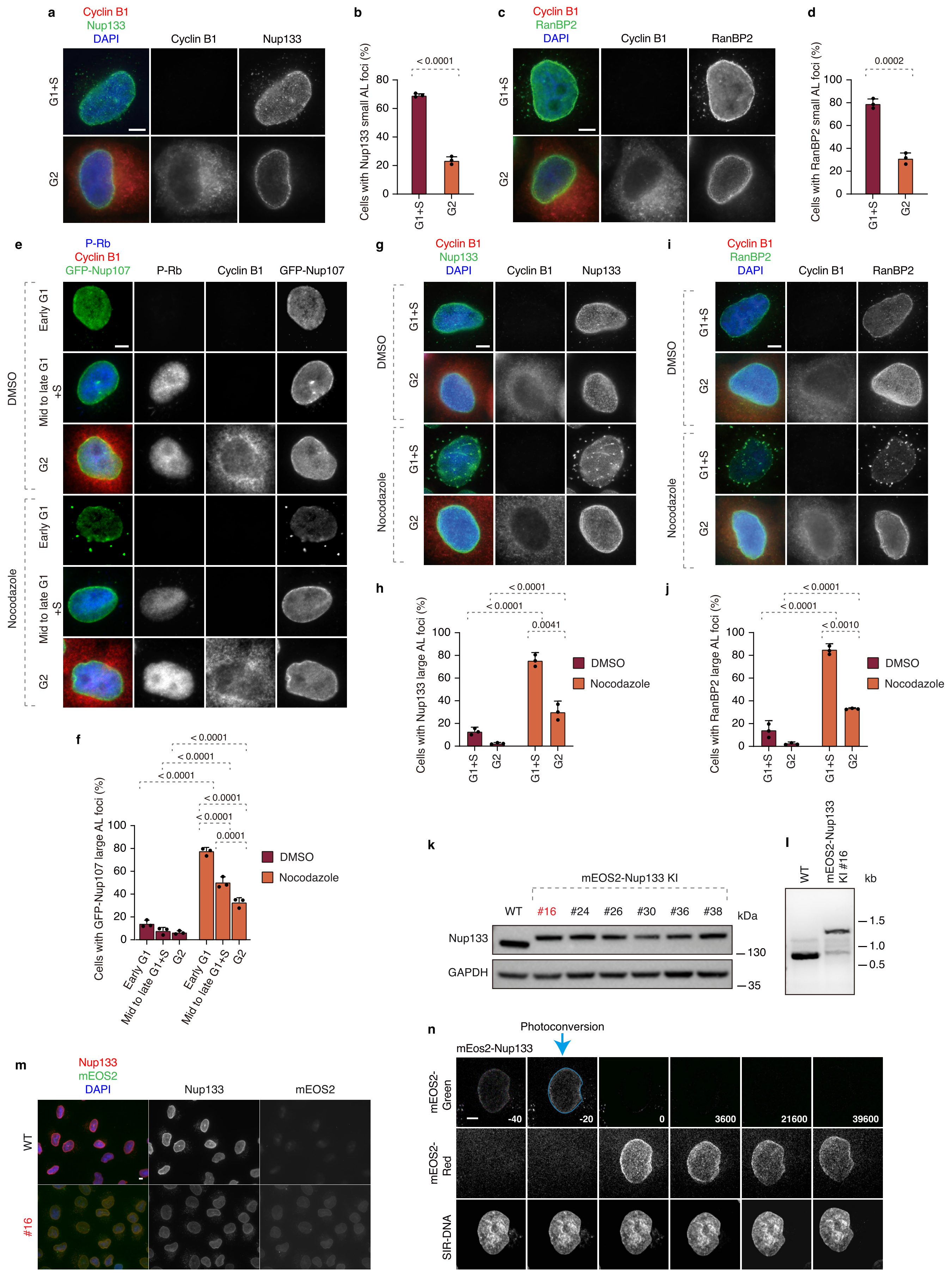
a
Cyclin B1
Nup133
DAPI
Cyclin B1
Nup133
G1+S
G2
b
Cells with Nup133 small AL foci (%)
< 0.0001
G1+S
G2
c
Cyclin B1
RanBP2
DAPI
Cyclin B1
RanBP2
G1+S
G2
d
Cells with RanBP2 small AL foci (%)
0.0002
G1+S
G2
e
P-Rb
Cyclin B1
GFP-Nup107
P-Rb
Cyclin B1
GFP-Nup107
DMSO
Nocodazole
Early G1
Mid to late G1 +S
G2
f
Cells with GFP-Nup107 large AL foci (%)
< 0.0001
0.0001
DMSO
Nocodazole
Early G1
Mid to late G1+S
G2
g
Cyclin B1
Nup133
DAPI
Cyclin B1
Nup133
DMSO
Nocodazole
G1+S
G2
h
Cells with Nup133 large AL foci (%)
< 0.0001
0.0041
DMSO
Nocodazole
G1+S
G2
i
Cyclin B1
RanBP2
DAPI
Cyclin B1
RanBP2
DMSO
Nocodazole
G1+S
G2
j
Cells with RanBP2 large AL foci (%)
< 0.0001
< 0.0010
DMSO
Nocodazole
G1+S
G2
k
mEOS2-Nup133 KI
WT
#16
#24
#26
#30
#36
#38
kDa
Nup133
GAPDH
130
35
l
WT
mEOS2-Nup133 KI #16
kb
1.5
1.0
0.5
m
Nup133
mEOS2
DAPI
Nup133
mEOS2
WT
#16
n
Photoconversion
mEos2-Nup133
mEOS2-Green
mEOS2-Red
SIR-DNA
-40
-20
0
3600
21600
39600

Supplementary Fig. 6. **AL foci are predominantly present in G1 and S cell cycle phases.** **a, b** Representative images of asynchronously proliferating HeLa cells co-labelled with anti-Nup133 (green) and anti-cyclin B1 (red) antibodies (a). The percentage of cells containing small Nup133-positive AL foci in cyclin B1-negative cells (G1 and S) and cyclin B1-positive cells (G2) was quantified (b); at least 200 cells per condition were analyzed (unpaired two-tailed t-test, mean ± SD, N = 3). Scale bars, 5 μm. Source data are provided as a Source Data file. **c, d** Representative images of asynchronously proliferating HeLa cells co-labelled with anti-RanBP2 (green) and anti-cyclin B1 (red) antibodies (c). The percentage of cells containing small RanBP2-positive AL foci in cyclin B1-negative cells (G1 and S) and cyclin B1-positive cells (G2) was quantified (d); at least 200 cells per condition were analyzed (unpaired two-tailed t-test, mean ± SD, N = 3). Scale bars, 5 μm. Source data are provided as a Source Data file. **e, f** Representative images of asynchronously proliferating 2xZFN-mEGFP-Nup107 HeLa cells treated with nocodazole (10 μM) or DMSO for 90 min and co-labelled with anti-p-Rb (blue) and anti-cyclin B1 (red) antibodies (e). The percentage of cells containing large AL foci in p-Rb-negative and cyclin B1-negative cells (early G1), p-Rb-positive and cyclin B1-negative cells (mid to late G1 and S), and p-Rb-positive and cyclin B1-positive cells (G2) was quantified (f); at least 200 cells per condition were analyzed (one-way ANOVA test, mean ± SD, N = 3). Scale bars, 5 μm. Source data are provided as a Source Data file. **g, h** Representative images of asynchronously proliferating HeLa cells treated with nocodazole (10 μM) or DMSO for 90 min and co-labelled with anti-Nup133 (green) and anti-cyclin B1 (red) antibodies (g). The percentage of cells containing large AL foci in cyclin B1-negative cells (G1 and S) and cyclin B1-positive cells (G2) was quantified (h); at least 200 cells per condition were analyzed (one-way ANOVA test, mean ± SD, N = 3). Scale bars, 5 μm. Source data are provided as a Source Data file. **i, j** Representative images of asynchronously proliferating HeLa cells treated with nocodazole (10 μM) or DMSO for 90 min and co-labelled with anti-RanBP2 (green) and anti-cyclin B1 (red) antibodies (i). The percentage of cells containing large AL foci in cyclin B1-negative cells (G1 and S) and cyclin B1-positive cells (G2) was quantified (j); at least 200 cells per condition were analyzed (one-way ANOVA test, mean ± SD, N = 3). Scale bars, 5 μm. Source data are provided as a Source Data file. **k, l** Validation of CRISPR-Cas9-mediated mEOS2-Nup133 knock-in (KI) HeLa cell clones by western blotting (k) and PCR (l). **m** Representative images of mEOS2-Nup133 KI HeLa cells co-labelled with DAPI (blue) and anti-Nup133 (red) antibodies. Scale bar, 5 μm. **n** Photoconversion of NE-localized Nup133 in mEOS2-Nup133 HeLa cells analyzed by live spinning-disk confocal microscopy. Selected representative frames are shown, with time indicated in seconds. Magnified boxed regions of the mEOS2-Red signal are shown in the lower panels. The blue dashed box indicates the photoconversion region, where mEOS2-Nup133 is converted from green to red fluorescence. Scale bar, 5 μm.

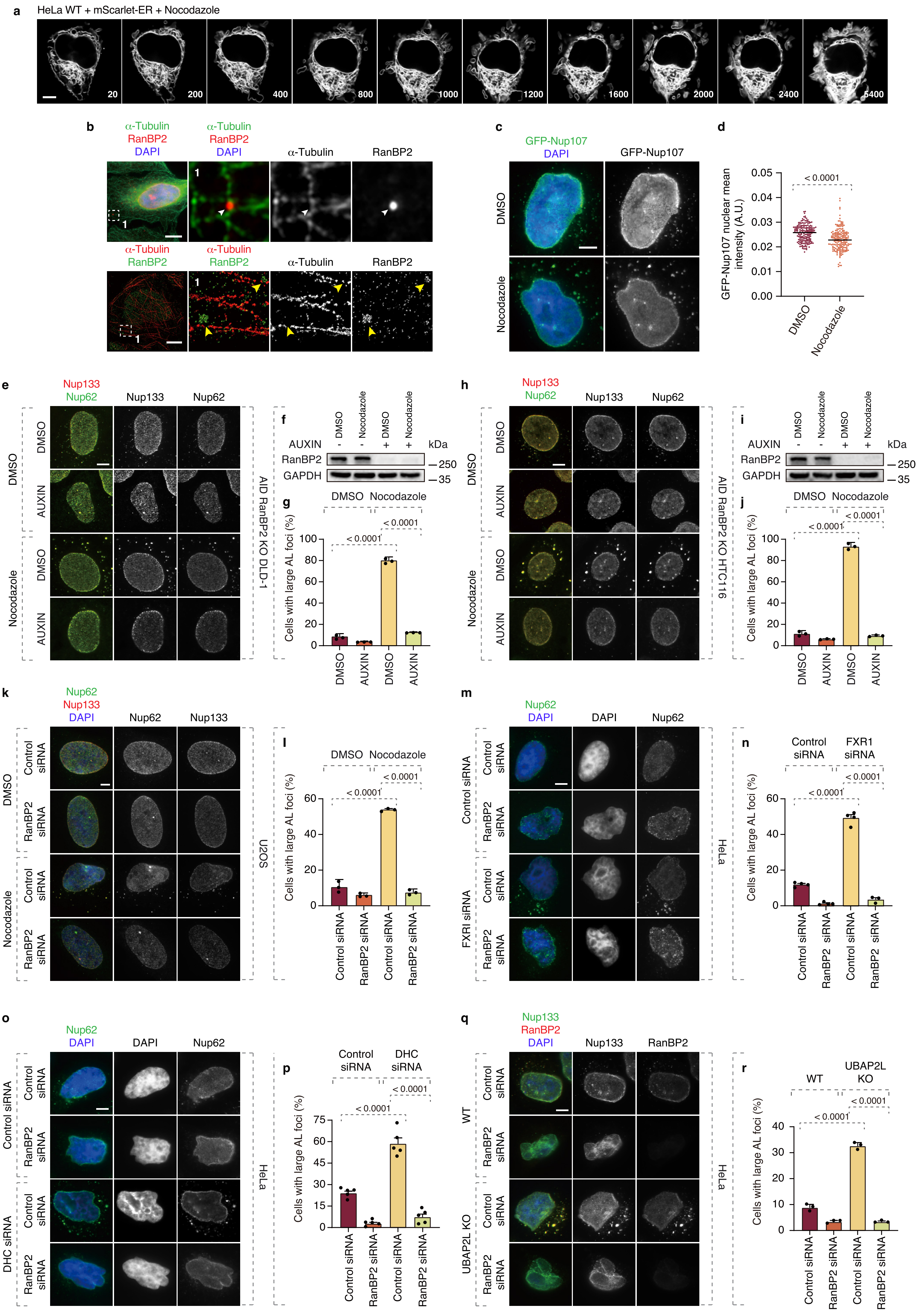
a
HeLa WT + mScarlet-ER + Nocodazole
20
200
400
800
1000
1200
1600
2000
2400
5400
b
α-Tubulin
RanBP2
DAPI
α-Tubulin
RanBP2
c
GFP-Nup107
DAPI
DMSO
Nocodazole
d
GFP-Nup107 nuclear mean intensity (A.U.)
< 0.0001
e
Nup133
Nup62
AUXIN
AID RanBP2 KO DLD-1
f
kDa
GAPDH
250
35
g
Cells with large AL foci (%)
h
AID RanBP2 KO HTC116
i
j
k
Control siRNA
RanBP2 siRNA
U2OS
l
m
FXRI siRNA
HeLa
n
FXR1 siRNA
o
DHC siRNA
p
q
WT
UBAP2L KO
r

Supplementary Fig. 7. **RanBP2 is required for the accumulation and clustering of large AL in challenged somatic cells. a** HeLa cells expressing the ER marker mScarlet-ER were treated with nocodazole (10 µM) and analyzed by live spinning-disk confocal microscopy. Selected representative frames are shown, with time indicated in seconds. Time 0 corresponds to nocodazole addition. Magnified boxed regions are shown in the upper left corner. Scale bar, 5 µm. **b** Representative immunofluorescence (IF) images and splitSMLM analyses showing co-localization of AL-NPCs with microtubules in WT HeLa cells. For IF (top), cells were co-labelled with anti-alpha tubulin (green), anti-RanBP2 (red), and DAPI. For splitSMLM (bottom), cells were co-labelled with anti-alpha tubulin (red) and anti-RanBP2 (green). Magnified boxed regions are shown in the corresponding numbered panels. Arrowheads indicate AL-NPCs localized near microtubules. Scale bars, IF, 5 µm; SMLM, 3 µm. **c, d** Representative images of 2xZFN-mEGFP-Nup107 HeLa cells treated with nocodazole (10 µM) or DMSO for 90 min (c). Cells were co-labelled with DAPI (blue). Nuclear envelope EGFP-Nup107 intensity was quantified (d); at least 200 cells per condition were analyzed (unpaired two-tailed t-test, mean ± SD, N = 3). Scale bars, 5 µm. Source data are provided as a Source Data file. **e-g** Representative images of auxin-inducible degron (AID)-RanBP2 knockout DLD-1 cells treated with auxin or DMSO for 4 h and subsequently with nocodazole (10 µM) or DMSO for 90 min (e). Cells were co-labelled with anti-Nup62 (red) and anti-Nup133 (green) antibodies. Western blot analysis of RanBP2 under the indicated conditions is shown (f). The percentage of cells containing large AL foci was quantified (g); at least 1000 cells per condition were analyzed (one-way ANOVA test, mean ± SD, N = 3). Scale bars, 5 µm. Source data are provided as a Source Data file. **h-j** Representative images of AID-RanBP2 knockout HCT116 cells treated with auxin or DMSO for 4 h and subsequently with nocodazole (10 µM) or DMSO for 90 min (h). Cells were co-labelled with anti-Nup62 (red) and anti-Nup133 (green) antibodies. Western blot analysis of RanBP2 under the indicated conditions is shown (i). The percentage of cells containing large AL foci was quantified (j); at least 1000 cells per condition were analyzed (one-way ANOVA test, mean ± SD, N = 3). Scale bars, 5 µm. Source data are provided as a Source Data file. **k, l** Representative images of U2OS cells treated with the indicated siRNAs for 48 h and subsequently with nocodazole (10 µM) or DMSO for 90 min (k). Cells were co-labelled with anti-Nup62 (green) and anti-Nup133 (red) antibodies. The percentage of cells containing large AL foci was quantified (l); at least 1000 cells per condition were analyzed (one-way ANOVA test, mean ± SD, N = 3). Scale bars, 5 µm. Source data are provided as a Source Data file. **m, n** Representative images of HeLa cells treated with the indicated siRNAs for 48 h (m). Cells were co-labelled with anti-Nup62 (green) and DAPI (blue). The percentage of cells containing large AL foci was quantified (n); at least 1000 cells per condition were analyzed (one-way ANOVA test, mean ± SD, N = 3). Scale bars, 5 µm. Source data are provided as a Source Data file. **o, p** Representative images of HeLa cells treated with the indicated siRNAs for 48 h (o). Cells were co-labelled with anti-Nup62 (green) and DAPI (blue). The percentage of cells containing large AL foci was quantified (p); at least 1000 cells per condition were analyzed (mean ± SD, N = 3). Scale bars, 5 µm. Source data are provided as a Source Data file. **q, r** Representative images of UBAP2L knockout and WT HeLa cells treated with the indicated siRNAs for 48 h (q). Cells were co-labelled with anti-Nup133 (green) and anti-RanBP2 (red) antibodies. The percentage of cells containing large AL foci was quantified (r); at least 1000 cells per condition were analyzed (one-way ANOVA test, mean ± SD, N = 3). Scale bars, 5 µm. Source data are provided as a Source Data file.

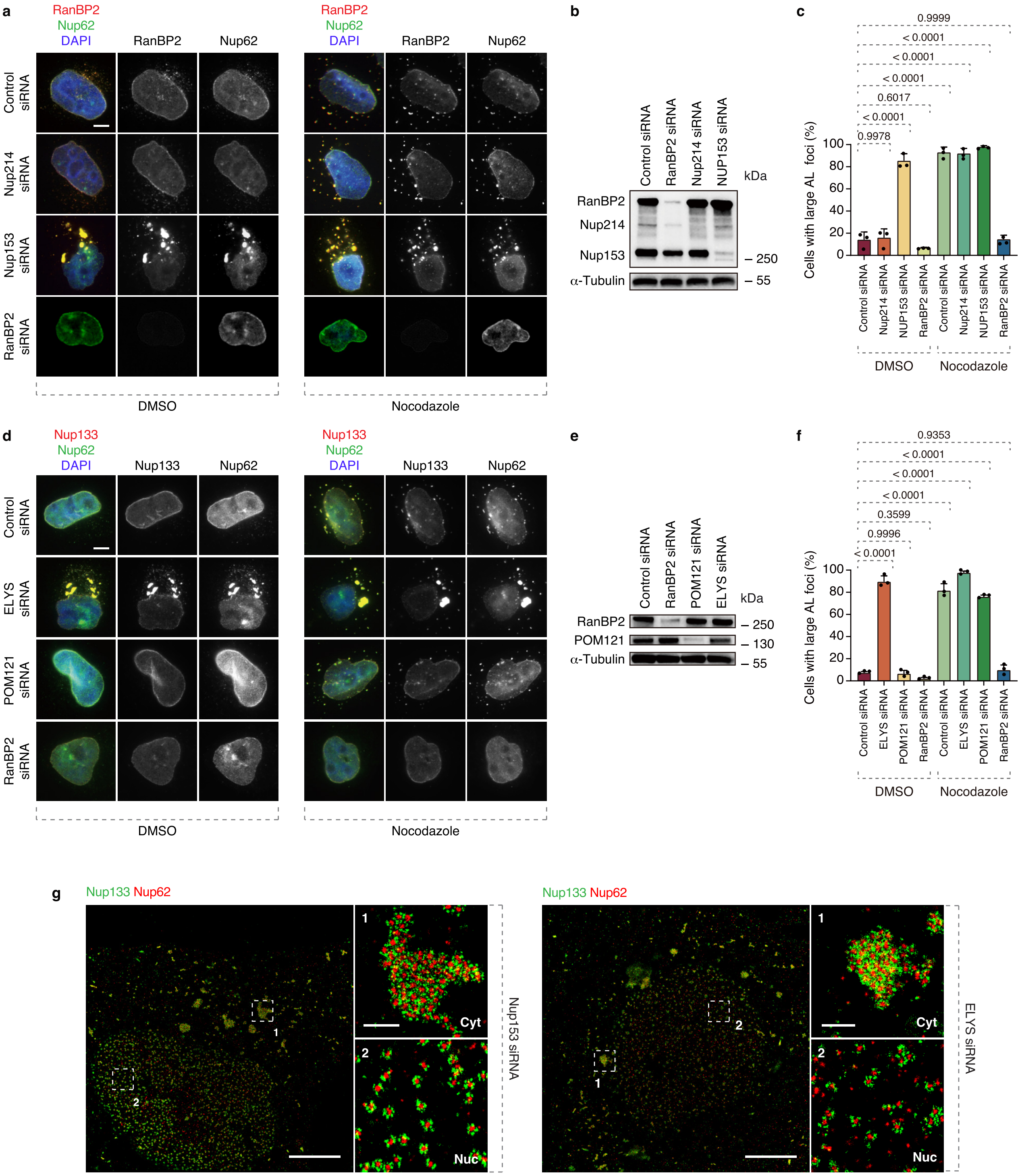
a
RanBP2
Nup62
DAPI
RanBP2
Nup62
Control siRNA
Nup214 siRNA
Nup153 siRNA
RanBP2 siRNA
DMSO
Nocodazole
b
Control siRNA
RanBP2 siRNA
Nup214 siRNA
NUP153 siRNA
kDa
RanBP2
Nup214
Nup153
α-Tubulin
– 250
– 55
c
Cells with large AL foci (%)
0.9999
< 0.0001
< 0.0001
< 0.0001
0.6017
< 0.0001
0.9978
Control siRNA
Nup214 siRNA
NUP153 siRNA
RanBP2 siRNA
DMSO
Nocodazole
d
Nup133
Nup62
DAPI
Nup133
Nup62
Control siRNA
ELYS siRNA
POM121 siRNA
RanBP2 siRNA
DMSO
Nocodazole
e
Control siRNA
RanBP2 siRNA
POM121 siRNA
ELYS siRNA
kDa
RanBP2
POM121
α-Tubulin
– 250
– 130
– 55
f
Cells with large AL foci (%)
0.9353
< 0.0001
< 0.0001
< 0.0001
0.3599
0.9996
< 0.0001
Control siRNA
ELYS siRNA
POM121 siRNA
RanBP2 siRNA
DMSO
Nocodazole
g
Nup133 Nup62
Cyt
Nuc
Nup153 siRNA
ELYS siRNA

Supplementary Fig. 8. **RanBP2, but not Nup153, Nup214, POM121 or ELYS, is required for the formation of large AL in challenged somatic cells. a-c** Representative images of HeLa cells treated with the indicated siRNAs for 48 h and subsequently with nocodazole (10 µM) or DMSO for 90 min (a). Cells were co-labelled with anti-Nup62 (green) and anti-RanBP2 (red) antibodies. Western blot analysis of RanBP2, Nup214, and Nup153 under the indicated conditions is shown (b). The percentage of cells containing large AL foci was quantified (c); at least 1000 cells per condition were analyzed (one-way ANOVA test, mean ± SD, N = 3). Scale bars, 5 µm. Source data are provided as a Source Data file. **d-f** Representative images of HeLa cells treated with the indicated siRNAs for 48 h and subsequently with nocodazole (10 µM) or DMSO for 90 min (d). Cells were co-labelled with anti-Nup62 (green) and anti-Nup133 (red) antibodies. Western blot analysis of RanBP2 and POM121 under the indicated conditions is shown (e). The percentage of cells containing large AL foci was quantified (f); at least 1000 cells per condition were analyzed (one-way ANOVA test, mean ± SD, N = 3). Scale bars, 5 µm. Source data are provided as a Source Data file. **g** Representative splitSMLM images showing NPCs at the nuclear (Nuc) surface and AL-NPCs in the cytoplasm (Cyt) of HeLa cells treated with Nup153 or ELYS siRNAs for 48 h (N = 3). Nup133 labels the cytoplasmic and nuclear rings of NPCs, and Nup62 marks the central channel. Magnified boxed regions are shown in the corresponding numbered panels. Scale bars, 3 µm and 0.3 µm (zoom).

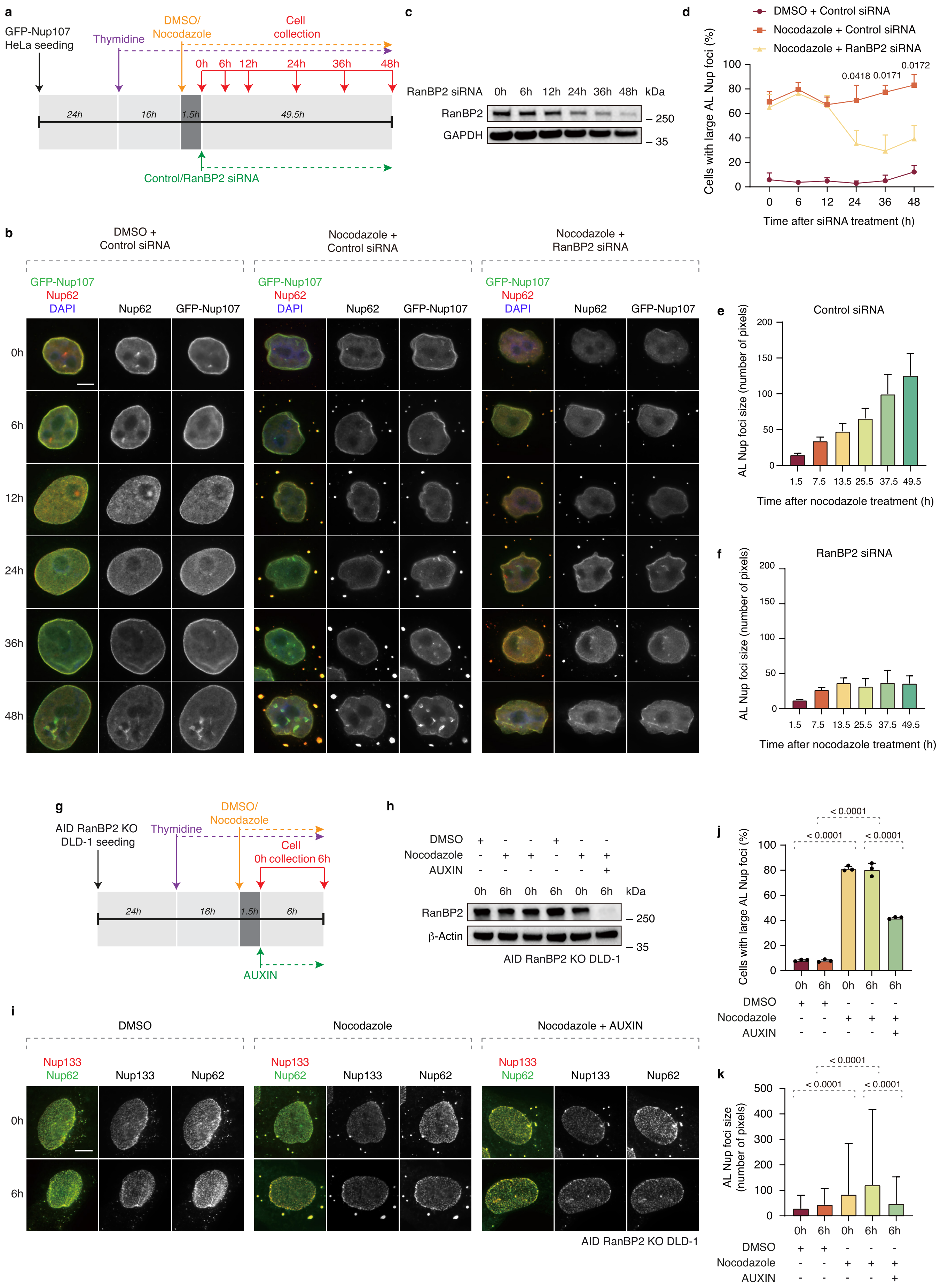

a
GFP-Nup107 HeLa seeding
Thymidine
DMSO/ Nocodazole
Cell collection
0h 6h 12h 24h 36h 48h
24h 16h 1.5h 49.5h
Control/RanBP2 siRNA
c
RanBP2 siRNA 0h 6h 12h 24h 36h 48h kDa
RanBP2 – 250
GAPDH – 35
d
DMSO + Control siRNA
Nocodazole + Control siRNA
Nocodazole + RanBP2 siRNA
Cells with large AL Nup foci (%)
0.0418 0.0171 0.0172
Time after siRNA treatment (h)
b
DMSO + Control siRNA
Nocodazole + Control siRNA
Nocodazole + RanBP2 siRNA
GFP-Nup107 Nup62 DAPI
Nup62
GFP-Nup107
0h 6h 12h 24h 36h 48h
e
Control siRNA
AL Nup foci size (number of pixels)
1.5 7.5 13.5 25.5 37.5 49.5
Time after nocodazole treatment (h)
f
RanBP2 siRNA
AL Nup foci size (number of pixels)
1.5 7.5 13.5 25.5 37.5 49.5
Time after nocodazole treatment (h)
g
AID RanBP2 KO DLD-1 seeding
Thymidine
DMSO/ Nocodazole
Cell 0h collection 6h
24h 16h 1.5h 6h
AUXIN
h
DMSO + - - + - -
Nocodazole - + + - + +
AUXIN - - - - - +
0h 6h 0h 6h 0h 6h kDa
RanBP2 – 250
β-Actin – 35
AID RanBP2 KO DLD-1
j
Cells with large AL Nup foci (%)
< 0.0001
< 0.0001
< 0.0001
0h 6h 0h 6h 6h
DMSO + + - - -
Nocodazole - - + + +
AUXIN - - - - +
i
DMSO
Nocodazole
Nocodazole + AUXIN
Nup133 Nup62
Nup133
Nup62
0h
6h
AID RanBP2 KO DLD-1
k
AL Nup foci size (number of pixels)
< 0.0001
< 0.0001
< 0.0001
0h 6h 0h 6h 6h
DMSO + + - - -
Nocodazole - - + + +
AUXIN - - - - +

Supplementary Fig. 9. **RanBP2 is required for the maintenance of large AL. a-f** Schematic of the experimental setup (a). HeLa cells were arrested in S phase by sustained thymidine treatment and subsequently treated with nocodazole (10 μM), transfected with the indicated siRNAs, and fixed at the indicated time points. Representative images are shown (b). Western blot analysis of RanBP2 at the indicated time points is shown (c). The percentage of cells containing large AL foci was quantified (d), and AL foci size was quantified (e, f). At least 200 cells and 1500 foci per condition were analyzed (one-way ANOVA test, mean ± SD, N = 3). Scale bars, 5 μm. Source data are provided as a Source Data file. **g-k** Schematic of the experimental setup (g). AID-RanBP2 knockout DLD-1 cells were arrested in S phase by sustained thymidine treatment and subsequently treated with nocodazole (10 μM) and auxin, and fixed at the indicated time points. Representative images are shown (i). Western blot analysis of RanBP2 under the indicated conditions is shown (h). The percentage of cells containing large AL foci was quantified (j), and AL foci size was quantified (k). At least 200 cells and 1500 foci per condition were analyzed (one-way ANOVA test, mean ± SD, N = 3). Scale bars, 5 μm. Source data are provided as a Source Data file.

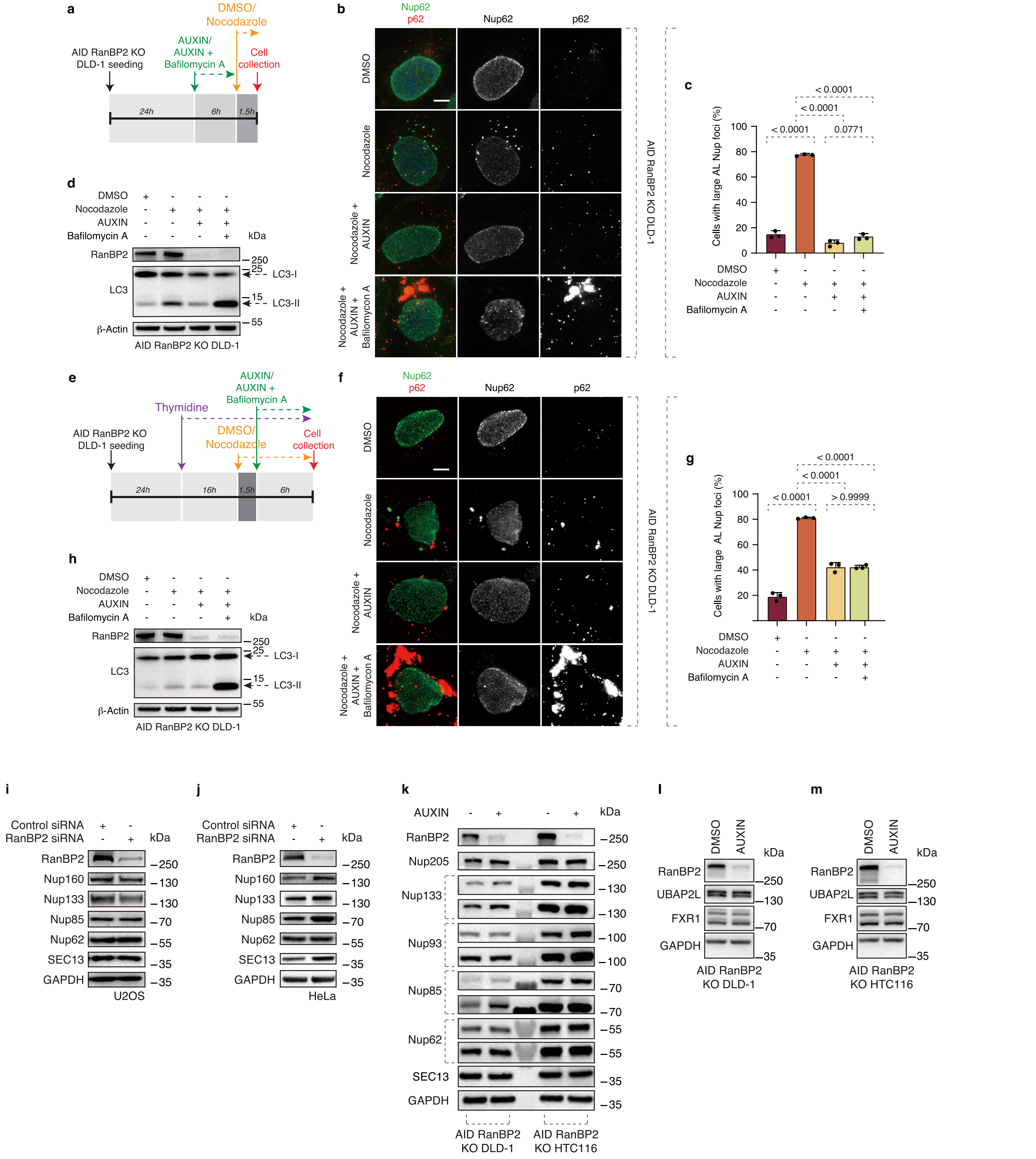

a
DMSO/ Nocodazole
AUXIN/ AUXIN + Bafilomycin A
AID RanBP2 KO DLD-1 seeding
Cell collection
24h
6h
1.5h
b
Nup62 p62
Nup62
p62
DMSO
Nocodazole
Nocodazole + AUXIN
Nocodazole + AUXIN + Bafilomycon A
AID RanBP2 KO DLD-1
c
Cells with large AL Nup foci (%)
< 0.0001
< 0.0001
< 0.0001
0.0771
DMSO
Nocodazole
AUXIN
Bafilomycin A
d
kDa
RanBP2
LC3
LC3-I
LC3-II
β-Actin
AID RanBP2 KO DLD-1
e
Thymidine
16h
f
g
> 0.9999
Cells with large AL Nup foci (%)
h
i
Control siRNA
RanBP2 siRNA
Nup160
Nup133
Nup85
Nup62
SEC13
GAPDH
U2OS
j
HeLa
k
AUXIN
Nup205
Nup93
AID RanBP2 KO DLD-1
AID RanBP2 KO HTC116
l
UBAP2L
FXR1
m
AID RanBP2 KO HTC116

Supplementary Fig. 10. **RanBP2-dependent formation and maintenance of large AL is independent of autophagy. a-d** Schematic of the experimental setup (a). AID-RanBP2 knockout DLD-1 cells were treated with nocodazole (10 μM), auxin, and bafilomycin at the indicated time points. Cells were co-labelled with anti-Nup62 (green) and anti-p62 (red) antibodies. Representative images are shown (b). The percentage of cells containing large AL foci was quantified (c). Western blot analysis of RanBP2 and LC3 under the indicated conditions is shown (d). At least 200 cells per condition were analyzed (one-way ANOVA test, mean ± SD, N = 3). Scale bars, 5 μm. Source data are provided as a Source Data file. **e-h** Schematic of the experimental setup (e). AID-RanBP3 knockout DLD-1 cells were arrested in S phase by sustained thymidine treatment and subsequently treated with nocodazole (10 μM), auxin, and bafilomycin at the indicated time points. Cells were co-labelled with anti-Nup62 (green) and anti-p62 (red) antibodies. Representative images are shown (f). The percentage of cells containing large AL foci was quantified (g). Western blot analysis of RanBP2 and LC3 under the indicated conditions is shown (h). At least 200 cells per condition were analyzed (one-way ANOVA test, mean ± SD, N = 3). Scale bars, 5 μm. Source data are provided as a Source Data file. **i-k** Protein levels of representative Nups were analyzed by western blotting in U2OS, HeLa, and AID-RanBP2 knockout DLD-1 or HCT116 cells treated with RanBP2 siRNA or auxin to induce RanBP2 depletion. **l, m** Protein levels of FXR1 and UBAP2L were analyzed by western blotting in AID-RanBP2 knockout DLD-1 or HCT116 cells treated with DMSO or auxin for 4 h to induce RanBP2 depletion and synchronized in early interphase.

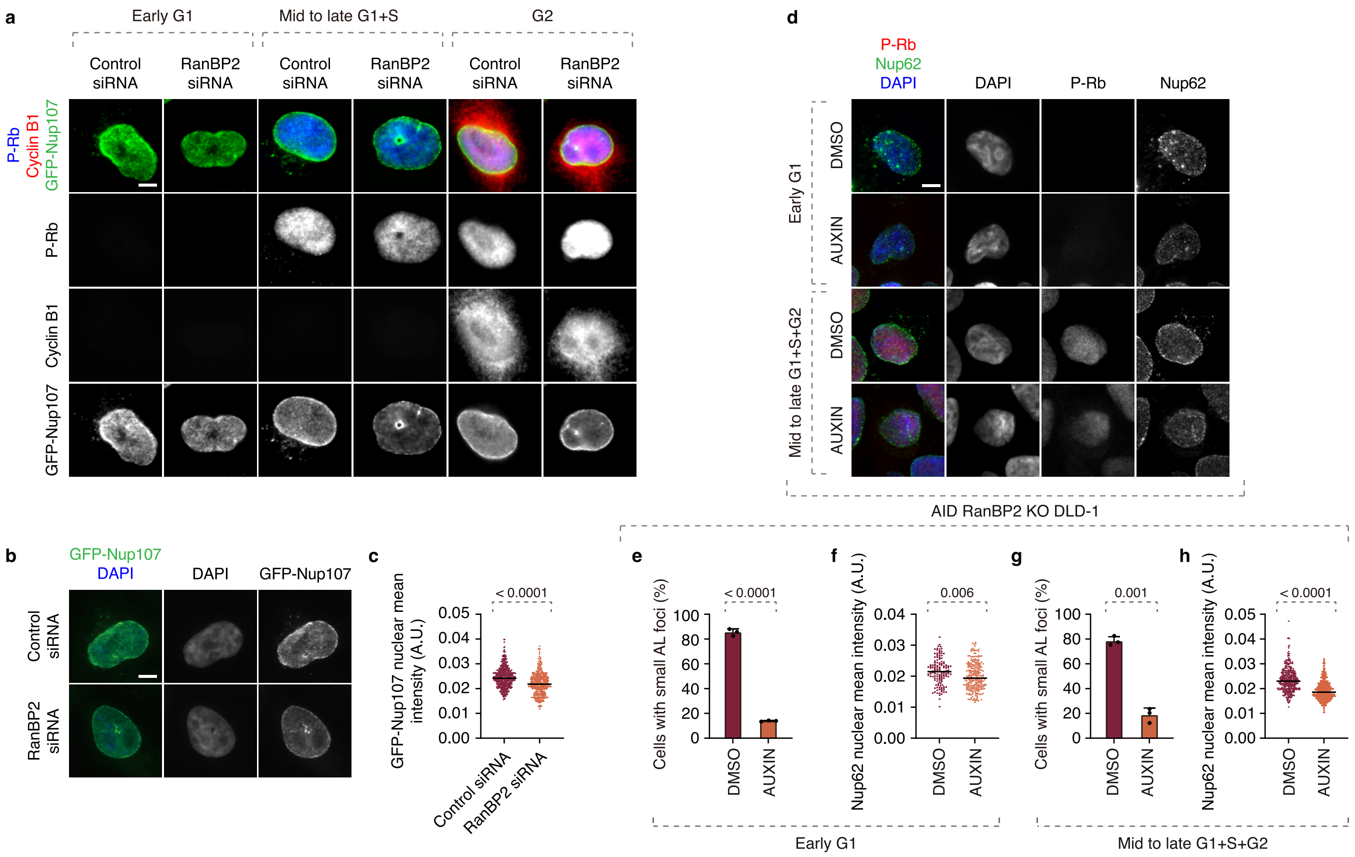

a
Early G1
Mid to late G1+S
G2
Control siRNA
RanBP2 siRNA
P-Rb
Cyclin B1
GFP-Nup107
b
GFP-Nup107
DAPI
Control siRNA
RanBP2 siRNA
c
GFP-Nup107 nuclear mean intensity (A.U.)
< 0.0001
d
P-Rb
Nup62
DAPI
DMSO
AUXIN
Early G1
Mid to late G1+S+G2
AID RanBP2 KO DLD-1
e
Cells with small AL foci (%)
< 0.0001
f
Nup62 nuclear mean intensity (A.U.)
0.006
g
0.001
h
< 0.0001
Early G1
Mid to late G1+S+G2

Supplementary Fig. 11. **RanBP2 is required for the biogenesis of small AL and localization of Nups to the NE in non-stressed interphasic cells. a** Representative images of asynchronously proliferating 2xZFN-mEGFP-Nup107 HeLa cells treated with the indicated siRNAs for 48 h and co-labelled with anti-p-Rb (blue) and anti-cyclin B1 (red) antibodies. The percentage of cells containing small AL foci in p-Rb-negative and cyclin B1-negative cells (early G1), p-Rb-positive and cyclin B1-negative cells (mid to late G1 and S), and p-Rb-positive and cyclin B1-positive cells (G2) was quantified in Fig. 4m. Scale bars, 5 µm. **b, c** Representative images of 2xZFN-mEGFP-Nup107 HeLa cells treated with RanBP2 siRNA for 48 h (b). Cells were co-labelled with DAPI (blue). Nuclear envelope GFP-Nup107 intensity was quantified (c). At least 400 cells per condition were analyzed (unpaired two-tailed t-test, mean ± SD, N = 3). Scale bars, 5 µm. Source data are provided as a Source Data file. **d-h** Representative immunofluorescence images showing Nup62 localization in AID-RanBP2 knockout DLD-1 cells at different cell cycle stages. p-Rb staining was used to distinguish early G1 cells (p-Rb-negative) from mid to late G1, S, and G2 cells (p-Rb-positive). Nuclei were stained with DAPI (d). The percentage of cells containing small AL foci in early G1 (e) and in mid to late G1, S, and G2 (g) was quantified; at least 300 cells per condition were analyzed (unpaired two-tailed t-test, mean ± SD, N = 3 independent experiments). Nuclear envelope Nup62 intensity in early G1 cells (f) and in mid to late G1, S, and G2 cells (h) was quantified; at least 150 cells per condition were analyzed (unpaired two-tailed t-test, mean ± SD, N = 3). Scale bars, 5 µm. Source data are provided as a Source Data file.

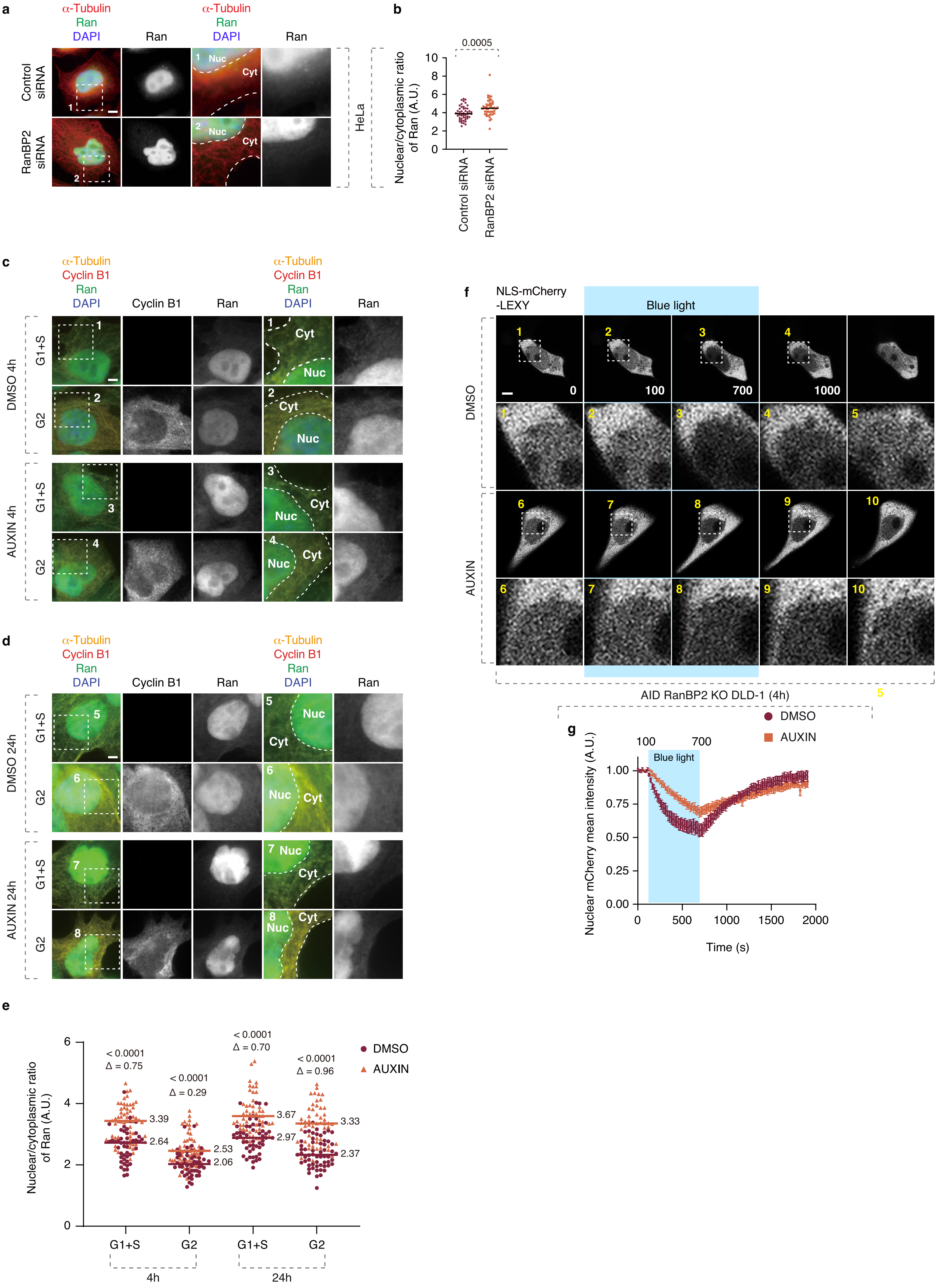

a
α-Tubulin
Ran
DAPI
Ran
Control siRNA
RanBP2 siRNA
Nuc
Cyt
HeLa
b
0.0005
Nuclear/cytoplasmic ratio of Ran (A.U.)
Control siRNA
RanBP2 siRNA
c
α-Tubulin
Cyclin B1
Ran
DAPI
Cyclin B1
Ran
DMSO 4h
AUXIN 4h
G1+S
G2
d
DMSO 24h
AUXIN 24h
e
Nuclear/cytoplasmic ratio of Ran (A.U.)
< 0.0001
Δ = 0.75
< 0.0001
Δ = 0.29
< 0.0001
Δ = 0.70
< 0.0001
Δ = 0.96
3.39
2.64
2.53
2.06
3.67
2.97
3.33
2.37
DMSO
AUXIN
G1+S
G2
4h
24h
f
NLS-mCherry -LEXY
Blue light
DMSO
AUXIN
0
100
700
1000
AID RanBP2 KO DLD-1 (4h)
g
DMSO
AUXIN
100
700
Blue light
Nuclear mCherry mean intensity (A.U.)
Time (s)

Supplementary Fig. 12. **RanBP2 supports nucleocytoplasmic transport. a, b** Representative immunofluorescence images showing nuclear (Nuc) and cytoplasmic (Cyt) localization of Ran in asynchronously proliferating WT and RanBP2 knockdown HeLa cells treated with RanBP2 siRNA for 48 h (a). Cells were co-labelled with anti-Ran (green), anti-alpha-tubulin (red), and DAPI. Magnified boxed regions are shown in the corresponding numbered panels. The nuclear-to-cytoplasmic (N/C) ratio of Ran was quantified (b). At least 100 cells per condition were analyzed (unpaired two-tailed t-test, mean ± SD, N = 3). Scale bar, 5 μm. Source data are provided as a Source Data file. **c-e** Representative immunofluorescence images showing nuclear (Nuc) and cytoplasmic (Cyt) localization of Ran in asynchronously proliferating AID-RanBP2 knockout DLD-1 cells treated with DMSO or auxin for 4 h or 24 h to induce RanBP2 depletion (c, e). Cells were co-labelled with anti-Ran (green), anti-alpha-tubulin-647 (yellow), anti-cyclin B1 antibodies, and DAPI. Magnified boxed regions are shown in the corresponding numbered panels. The nuclear-to-cytoplasmic (N/C) ratio of Ran was quantified (e). At least 100 cells per condition were analyzed (unpaired two-tailed t-test, mean ± SD, N = 3). Scale bar, 5 μm. Source data are provided as a Source Data file. **f, g** Representative immunofluorescence images showing nuclear fluorescence of the NLS-mCherry-LEXY shuttling reporter expressed in AID-RanBP2 knockout DLD-1 cells treated with DMSO or auxin for 4 h to induce RanBP2 depletion (f). Cells were incubated in the dark for 100 s, followed by blue light irradiation for 600 s and a 1200 s dark-recovery phase. Time is indicated in seconds. Relative nuclear fluorescence was quantified (g). At least 10 cells per condition were analyzed (mean ± SD, N = 3). Source data are provided as a Source Data file.

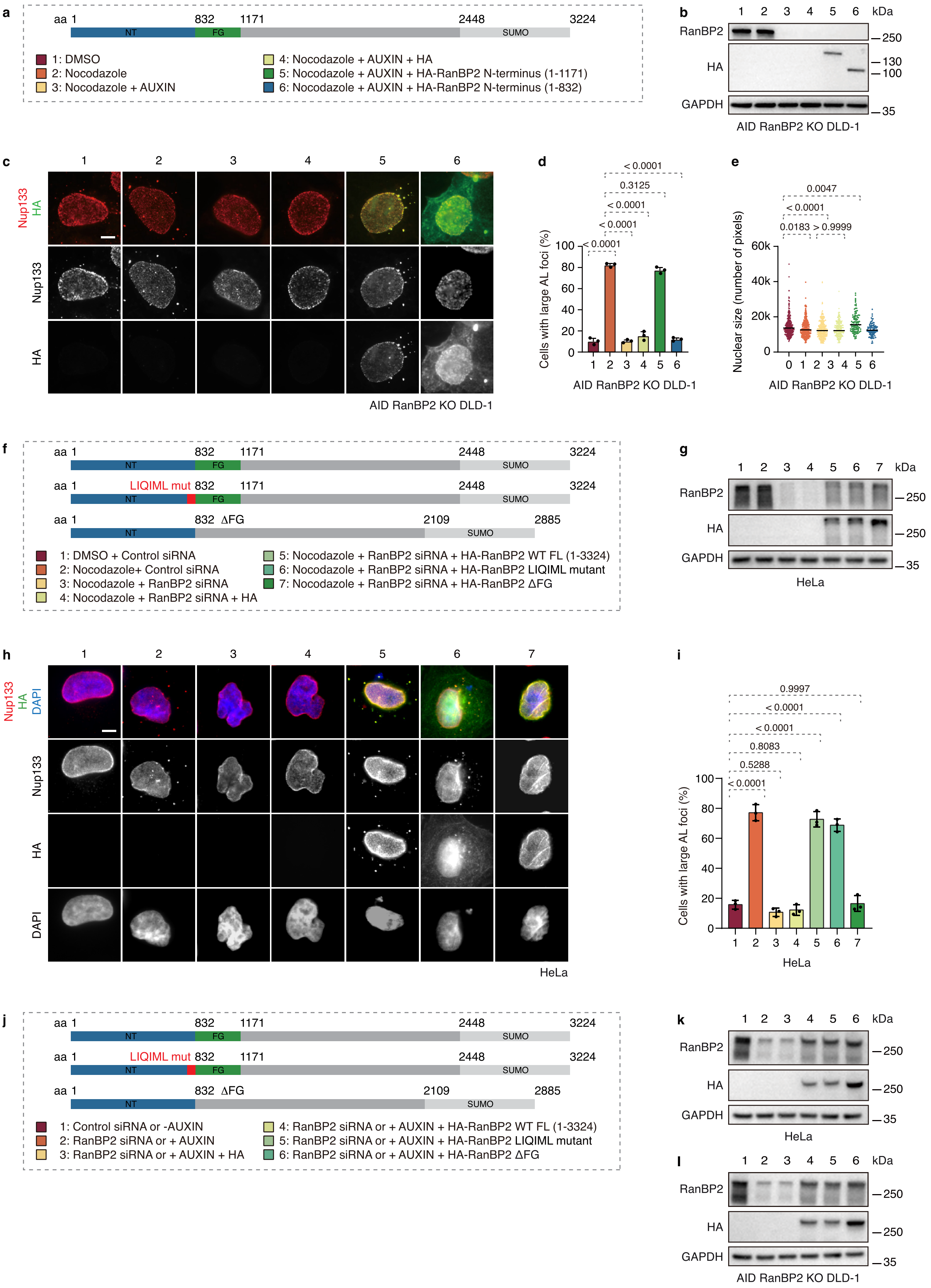
a
aa 1
832
1171
2448
3224
NT
FG
SUMO
1: DMSO
2: Nocodazole
3: Nocodazole + AUXIN
4: Nocodazole + AUXIN + HA
5: Nocodazole + AUXIN + HA-RanBP2 N-terminus (1-1171)
6: Nocodazole + AUXIN + HA-RanBP2 N-terminus (1-832)
b
RanBP2
HA
GAPDH
kDa
250
130
100
35
AID RanBP2 KO DLD-1
c
Nup133
HA
AID RanBP2 KO DLD-1
d
Cells with large AL foci (%)
< 0.0001
0.3125
< 0.0001
< 0.0001
< 0.0001
AID RanBP2 KO DLD-1
e
Nuclear size (number of pixels)
0.0047
< 0.0001
0.0183
> 0.9999
60k
40k
20k
AID RanBP2 KO DLD-1
f
LIQIML mut
832 ΔFG
2109
2885
1: DMSO + Control siRNA
2: Nocodazole+ Control siRNA
3: Nocodazole + RanBP2 siRNA
4: Nocodazole + RanBP2 siRNA + HA
5: Nocodazole + RanBP2 siRNA + HA-RanBP2 WT FL (1-3324)
6: Nocodazole + RanBP2 siRNA + HA-RanBP2 LIQIML mutant
7: Nocodazole + RanBP2 siRNA + HA-RanBP2 ΔFG
g
HeLa
h
Nup133
HA
DAPI
HeLa
i
Cells with large AL foci (%)
0.9997
< 0.0001
< 0.0001
0.8083
0.5288
< 0.0001
HeLa
j
1: Control siRNA or -AUXIN
2: RanBP2 siRNA or + AUXIN
3: RanBP2 siRNA or + AUXIN + HA
4: RanBP2 siRNA or + AUXIN + HA-RanBP2 WT FL (1-3324)
5: RanBP2 siRNA or + AUXIN + HA-RanBP2 LIQIML mutant
6: RanBP2 siRNA or + AUXIN + HA-RanBP2 ΔFG
k
HeLa
l
AID RanBP2 KO DLD-1

Supplementary Fig. 13. **RanBP2 is specifically required for the formation of large AL through its N-terminal FG repeat region. a-e** Rescue experiments using different RanBP2 protein fragments (a). Western blot analysis of RanBP2 fragments under the indicated conditions is shown (b). Representative images of AID-RanBP2 knockout DLD-1 cells treated with DMSO or auxin, transfected with the indicated RanBP2 fragments, and subsequently treated with nocodazole (10 μM) or DMSO for 90 min are shown (c). Cells were co-labelled with anti-Nup133 (red) and anti-HA (green) antibodies. The percentage of cells containing large AL foci was quantified (d), and nuclear size was quantified (e). At least 300 cells per condition were analyzed (one-way ANOVA test, mean ± SD, N = 3). Scale bar, 5 μm. Source data are provided as a Source Data file. **f-i** Rescue experiments using different full-length RanBP2 protein versions (f). Western blot analysis of RanBP2 versions under the indicated conditions is shown (g), with fragment numbering corresponding to the schematic. Representative images of HeLa cells treated with control or RanBP2 siRNAs, transfected with the indicated RanBP2 versions, and subsequently treated with nocodazole (10 μM) or DMSO for 90 min are shown (h). Cells were co-labelled with anti-Nup133 (red) and anti-HA (green) antibodies. The percentage of cells containing large AL foci was quantified (i). At least 300 cells per condition were analyzed (one-way ANOVA test, mean ± SD, N = 3). Scale bar, 5 μm. Source data are provided as a Source Data file. **j-l** Rescue experiments using different RanBP2 protein versions (j). Western blot analysis of RanBP2 fragments under the indicated conditions is shown (k, l), with fragment numbering corresponding to the schematic. Representative images and quantitative analyses are shown in Fig. 5j and 5k.

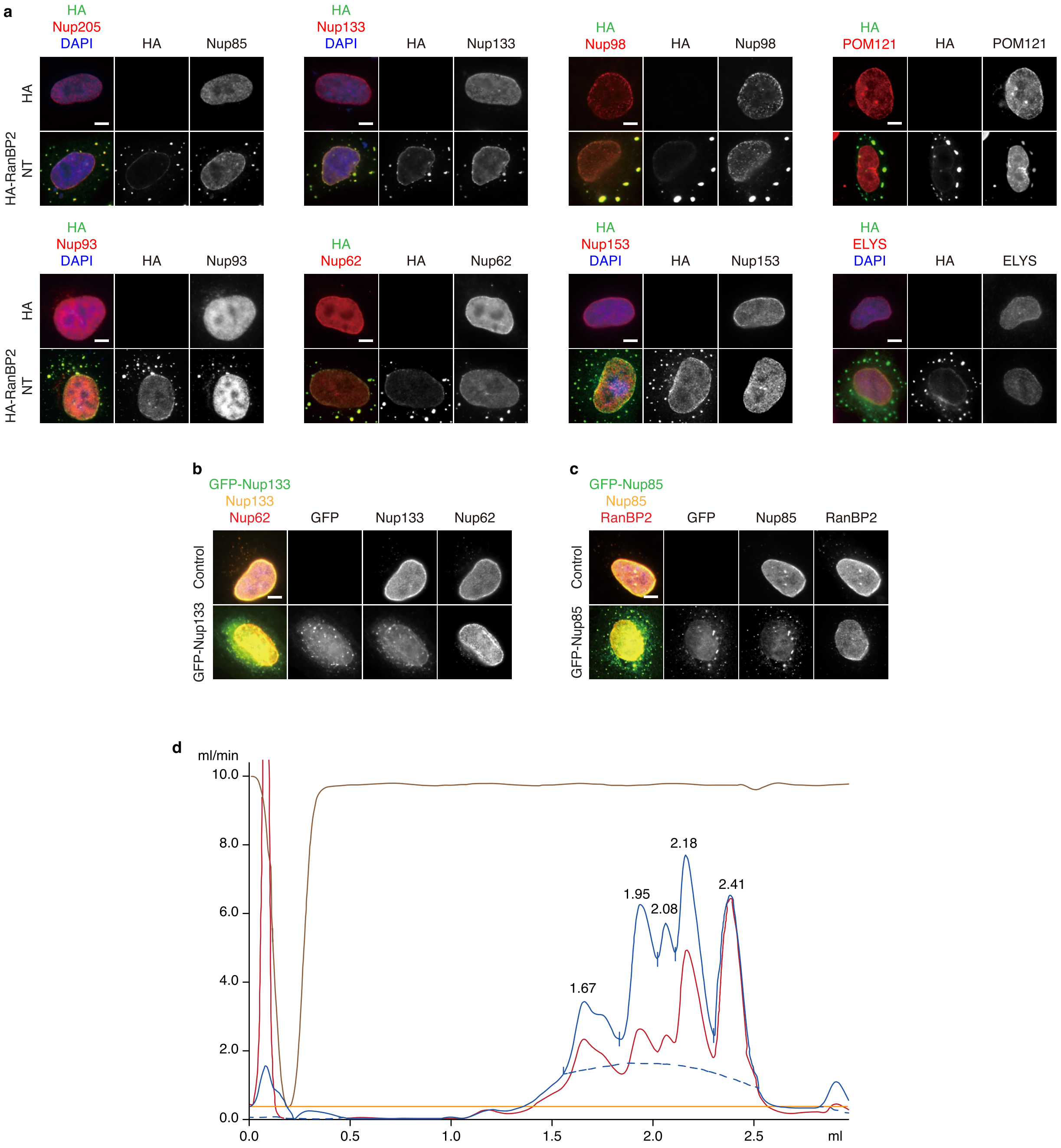

Component	Stoke's radius (A)	MW (Da)	Log MW	Volume (ml)
**Thyroglobulin (bovine)**	**86**	670,000	5.826	
**g**-globulin (bovine)	**51**	158,000	5.198	1.95
**Ovalbumin (chicken)**	**28**	44,000	4.643	2.08
**Myoglobulin (horse)**	**19**	17,000	4.23	2.18
**Vitamin B12**	**8.5**	1,350	3.13	2.41

Supplementary Fig. 14. **N-terminal fragment of RanBP2 promotes AL formation. a** Representative images of HeLa cells transfected with HA or HA-RanBP2 N-terminal (NT) fragment and labelled for Nups from different NPC subcomplexes, as indicated (N = 3). Cells were co-labelled with DAPI (blue), anti-Nup antibodies (red), and anti-HA (green). Scale bars, 5 µm. **b** Representative images of HeLa cells transfected with GFP-Nup133. Cells were co-labelled with anti-Nup133 (yellow) and anti-Nup62 (red) antibodies. Scale bars, 5 µm. **c** Representative images of HeLa cells transfected with GFP-Nup85. Cells were co-labelled with anti-Nup85 (yellow) and anti-RanBP2 (red) antibodies. Scale bars, 5 µm. Note that expression of RanBP2-NT, but not Nup133 or Nup85, induces large AL foci in which multiple Nups co-localize. **d** Calibration of the Superose 6 size-exclusion column using thyroglobulin (bovine), gamma-globulin (bovine), ovalbumin (chicken), myoglobin (horse), and vitamin B12. The calibration profile and molecular weights of the protein standards are indicated.

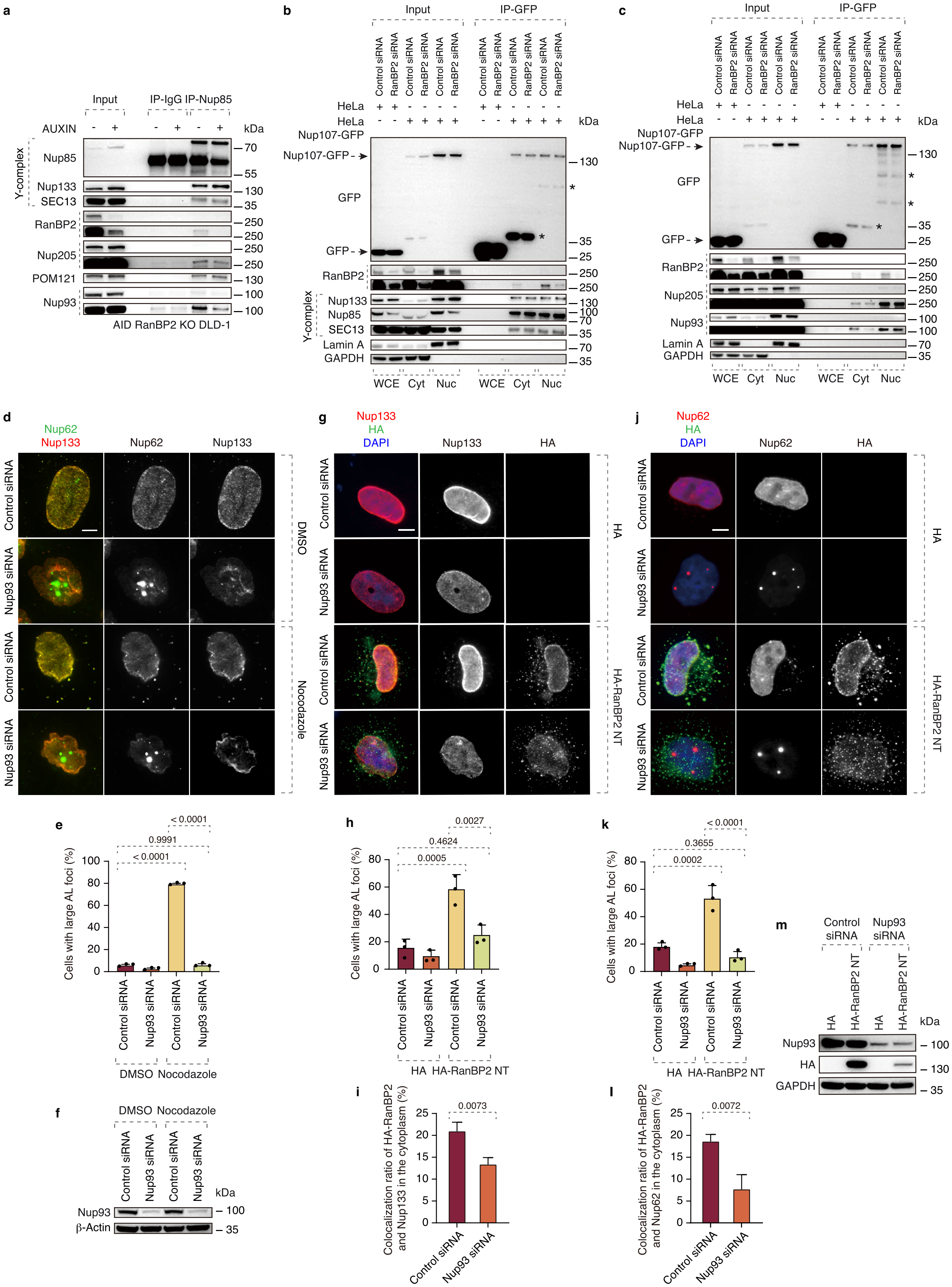

a
Input
IP-IgG IP-Nup85
AUXIN
kDa
Y-complex
Nup85
Nup133
SEC13
RanBP2
Nup205
POM121
Nup93
AID RanBP2 KO DLD-1
b
Input
IP-GFP
Control siRNA
RanBP2 siRNA
HeLa
HeLa Nup107-GFP
Nup107-GFP
GFP
RanBP2
Nup133
Nup85
SEC13
Lamin A
GAPDH
WCE Cyt Nuc
c
Input
IP-GFP
HeLa
HeLa Nup107-GFP
Nup107-GFP
GFP
RanBP2
Nup205
Nup93
Lamin A
GAPDH
WCE Cyt Nuc
d
Nup62
Nup133
Control siRNA
Nup93 siRNA
DMSO
Nocodazole
g
Nup133
HA
DAPI
HA
HA-RanBP2 NT
j
Nup62
HA
DAPI
e
Cells with large AL foci (%)
< 0.0001
0.9991
< 0.0001
DMSO Nocodazole
f
DMSO Nocodazole
Nup93
β-Actin
h
0.0027
0.4624
0.0005
HA HA-RanBP2 NT
i
Colocalization ratio of HA-RanBP2 and Nup133 in the cytoplasm (%)
0.0073
Control siRNA
Nup93 siRNA
k
< 0.0001
0.3655
0.0002
l
Colocalization ratio of HA-RanBP2 and Nup62 in the cytoplasm (%)
0.0072
m
Control siRNA
Nup93 siRNA
HA
HA-RanBP2 NT
Nup93
HA
GAPDH
kDa

Supplementary Fig. 15. **RanBP2 drives cytosolic AL-NPC assembly by promoting the interaction between Nup93 and the Y complex. a** AID-RanBP2 knockout DLD-1 cells were treated with auxin to induce RanBP2 depletion. Cell lysates were immunoprecipitated using anti-Nup85 antibodies or IgG and analyzed by western blotting (at least 3 independent experiments). **b, c** Cytoplasmic or nuclear extracts of HeLa cells expressing GFP or 2xZFN-mEGFP-Nup107 were immunoprecipitated using GFP-Trap A beads and analyzed by western blotting (at least 3 independent experiments). **d-f** Representative images of HeLa cells treated with the indicated siRNAs for 48 h and subsequently with nocodazole (10 μM) or DMSO for 90 min (d). Cells were co-labelled with anti-Nup62 (green) and anti-Nup133 (red) antibodies. The percentage of cells containing large AL foci was quantified (e). At least 1000 cells per condition were analyzed (one-way ANOVA test, mean ± SD, N = 3). Western blot analysis of Nup93 under the indicated conditions is shown (f). Scale bars, 5 μm. Source data are provided as a Source Data file. **g-m** Representative images of HeLa cells treated with the indicated siRNAs and transfected with HA or HA-RanBP2-NT fragment. Cells were co-labelled with anti-Nup133 (red), anti-HA (green), and DAPI (blue) (g), or with anti-Nup62 (red), anti-HA (green), and DAPI (blue) (j). Non-AL Nup62 foci lacking Nup133 were observed in the nucleus (g, j). The percentage of cells containing large AL foci was quantified (h, k), and the co-localization ratio of HA-RanBP2-NT with Nup62 was quantified (i, l). At least 300 cells per condition were analyzed (unpaired two-tailed t-test or one-way ANOVA test, mean ± SD, N = 3). Western blot analysis of Nup93 and HA-RanBP2-NT under the indicated conditions is shown (m). Scale bars, 5 μm. Source data are provided as a Source Data file.

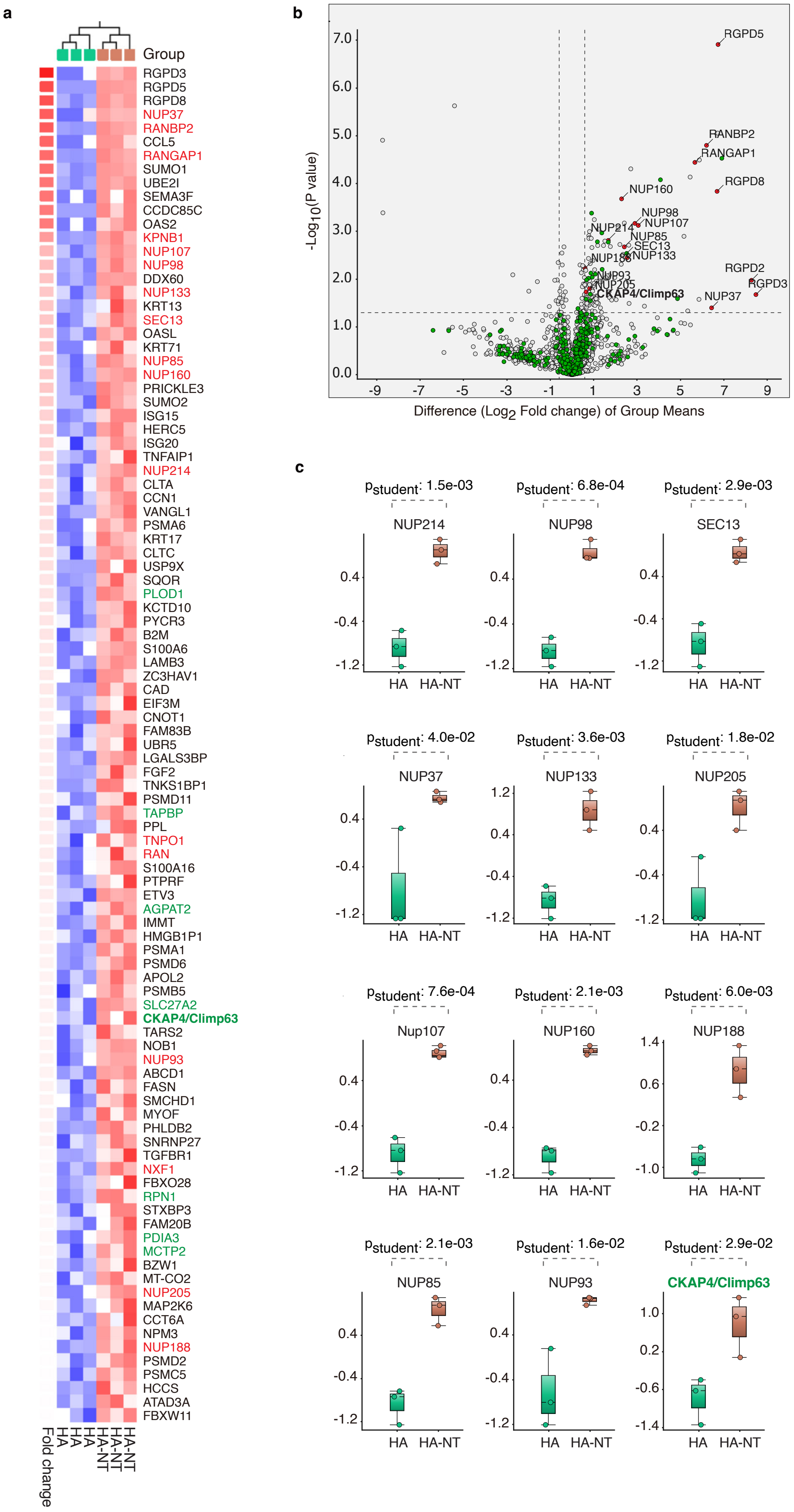
a
Group
RGPD3
RGPD5
RGPD8
NUP37
RANBP2
CCL5
RANGAP1
SUMO1
UBE2I
SEMA3F
CCDC85C
OAS2
KPNB1
NUP107
NUP98
DDX60
NUP133
KRT13
SEC13
OASL
KRT71
NUP85
NUP160
PRICKLE3
SUMO2
ISG15
HERC5
ISG20
TNFAIP1
NUP214
CLTA
CCN1
VANGL1
PSMA6
KRT17
CLTC
USP9X
SQOR
PLOD1
KCTD10
PYCR3
B2M
S100A6
LAMB3
ZC3HAV1
CAD
EIF3M
CNOT1
FAM83B
UBR5
LGALS3BP
FGF2
TNKS1BP1
PSMD11
TAPBP
PPL
TNPO1
RAN
S100A16
PTPRF
ETV3
AGPAT2
IMMT
HMGB1P1
PSMA1
PSMD6
APOL2
PSMB5
SLC27A2
CKAP4/Climp63
TARS2
NOB1
NUP93
ABCD1
FASN
SMCHD1
MYOF
PHLDB2
SNRNP27
TGFBR1
NXF1
FBXO28
RPN1
STXBP3
FAM20B
PDIA3
MCTP2
BZW1
MT-CO2
NUP205
MAP2K6
CCT6A
NPM3
NUP188
PSMD2
PSMC5
HCCS
ATAD3A
FBXW11
Fold change
HA
HA
HA
HA-NT
HA-NT
HA-NT
b
-Log10(P value)
Difference (Log2 Fold change) of Group Means
RGPD5
RANBP2
RANGAP1
RGPD8
NUP160
NUP98
NUP107
NUP214
NUP85
SEC13
NUP188
NUP133
NUP93
NUP205
CKAP4/Climp63
RGPD2
RGPD3
NUP37
c
pstudent: 1.5e-03
NUP214
pstudent: 6.8e-04
NUP98
pstudent: 2.9e-03
SEC13
pstudent: 4.0e-02
NUP37
pstudent: 3.6e-03
NUP133
pstudent: 1.8e-02
NUP205
pstudent: 7.6e-04
Nup107
pstudent: 2.1e-03
NUP160
pstudent: 6.0e-03
NUP188
pstudent: 2.1e-03
NUP85
pstudent: 1.6e-02
NUP93
pstudent: 2.9e-02
CKAP4/Climp63
HA
HA-NT

Supplementary Fig. 16. **RanBP2-dependent AL interactome. a-c** Liquid chromatography-mass spectrometry (LC-MS) was used to identify proteins interacting with HA-tagged RanBP2 N-terminal (NT) fragment, with the HA tag alone used as a negative control. A heatmap shows proteins displaying a greater than 1.5-fold change with a p-value < 0.05 between the two groups (a). A volcano plot comparing the two conditions is shown (b), using cutoffs of fold change > 1.5 and FDR < 0.05 to identify differentially interacting proteins. NPC-related proteins are highlighted in red and ER-related factors in green. Box plots of selected Nups and Climp63 (CKAP4) are shown (c).

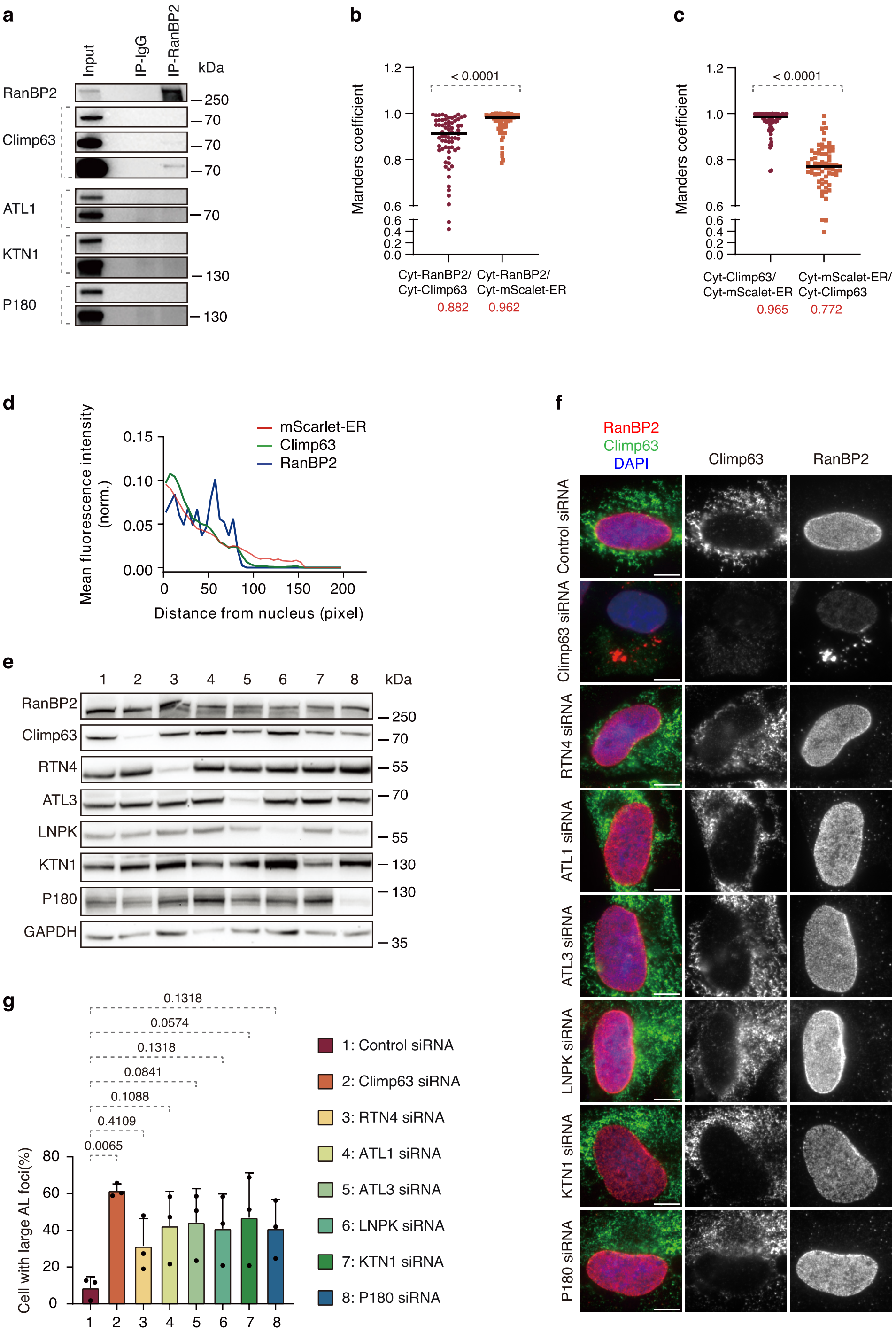
a
Input
IP-IgG
IP-RanBP2
kDa
RanBP2
Climp63
ATL1
KTN1
P180
250
70
130
b
Manders coefficient
< 0.0001
Cyt-RanBP2/
Cyt-Climp63
Cyt-RanBP2/
Cyt-mScalet-ER
0.882
0.962
c
Cyt-Climp63/
Cyt-mScalet-ER
Cyt-mScalet-ER/
Cyt-Climp63
0.965
0.772
d
Mean fluorescence intensity
(norm.)
mScarlet-ER
Climp63
RanBP2
Distance from nucleus (pixel)
e
1 2 3 4 5 6 7 8
RanBP2
Climp63
RTN4
ATL3
LNPK
KTN1
P180
GAPDH
250
70
55
130
35
f
RanBP2
Climp63
DAPI
Control siRNA
Climp63 siRNA
RTN4 siRNA
ATL1 siRNA
ATL3 siRNA
LNPK siRNA
KTN1 siRNA
P180 siRNA
g
0.1318
0.0574
0.1318
0.0841
0.1088
0.4109
0.0065
Cell with large AL foci(%)
1: Control siRNA
2: Climp63 siRNA
3: RTN4 siRNA
4: ATL1 siRNA
5: ATL3 siRNA
6: LNPK siRNA
7: KTN1 siRNA
8: P180 siRNA

Supplementary Fig. 17. **Climp63 facilitates localization of AL-NPCs to ER sheets. a** HeLa cell lysates were immunoprecipitated using anti-RanBP2 antibodies or IgG and analyzed by western blotting (at least 3 independent experiments). **b-d** Co-localization of Climp63, mScarlet-ER, and RanBP2 in the cytoplasm was quantified (b, c) in HeLa cells expressing the ER marker mScarlet-ER. Representative images are shown in Fig. 7c. Intensity profiles as a function of distance from the nucleus for the three fluorescent channels are shown (d). 150 cells were analyzed (unpaired two-tailed t-test, mean ± SD). Source data are provided as a Source Data file. **e-g** MRC5 cells treated with siRNAs targeting the indicated ER-associated factors were analyzed by western blotting (e). Representative images show cells co-labelled with anti-RanBP2 (red), anti-Climp63 (green), and DAPI (blue) (f). The percentage of cells containing large AL foci was quantified (g). At least 300 cells per condition were analyzed (one-way ANOVA test, mean ± SD, N = 3). Scale bars, 5 μm. Source data are provided as a Source Data file.

Supplementary Table 1. Reagents used for cloning, siRNA, and CRISPR/Cas9 experiments

This table summarizes the cloning primers, siRNAs, and guide RNAs used in this study.

**PCR primers**	
**Name**	**Sequence**
RanBP2(1-832)-F	GGCCGGATCCGAATTCATGAGGCGCAGCAAGGCT
RanBP2(1-832)-R	CGATCTCGAGGAATTTTAGCTATTTAGTTTCAACTCCTGCAT T
RanBP2(1-832)-F-(FL without FG repeat)	GGCCGGATCCGAATTCATGAGGCGCAGCAAGGCT
RanBP2(1-832)-R-(FL without FG repeat)	TACAGGCTCAAAGTGGCTATTTAGTTTCAACTCCTGCATT
RanBP2(1171-3224)-F-(FL without FG repeat)	CACTTTGAGCCTGTAGTACC
RanBP2(1171-3224)-R-(FL without FG repeat)	CGATCTCGAGGAATTTTATATCTGTCCACATTCTGTGATAG
PUC57-NUP133U-F	gacggccagtgaattCAACTGGCCAGTTTTACCAAGC
NUP133U-R	GACTCCAAGGAGCAGCGACT
NUP133U-mCherry-F	AGTCGCTGCTCCTTGGAGTCatggtgagcaagggcgagga
mCherry-R	cttgtacagctcgtccatgc
mCherry-NUP133D-F	gcatggacgagctgtacaagATGTTCCCAGCCGCC
NUP133D-PUC57-R	taccgagctcgaattTTATGAACATGGTGTGTGTTGG
NUP133U-mEos2-F	AGTCGCTGCTCCTTGGAGTCatgagtgcgattaagccagac
mEos2-R	tcgtctggcattgtcaggcaat
mEos2-NUP133D-F	attgcctgacaatgccagacgaATGTTCCCAGCCGCC

RanBP2 siRNA-resistant-F	GGAAGAAAACACAGATAAGGCCGTTGAATGTTACAGGC
RanBP2 siRNA-resistant-R	GCCTGTAACATTCAACGGCCTTATCTGTGTTTTCTTCC
**siRNAs**	
**Targeted gene**	**Sequence or Catalogue number**
Non-targeting individual siRNA	5’-UAAGGCUAUGAAGAGAUAC-3’
FXR1	5’-AAACGGAAUCUGAGCGUAA-3’
UBAP2L	5'-CAACACAGCAGCACGUUAU-3'
RanBP2	5'-CACAGACAAAGCCGUUGAA-3'
Nup153	5’-GGACUUGUUAGAUCTAGUU-3’
ELYS	5’-AUUAUCUACAUAAUUGCUCUU-3’
POM121	Life Technologies 4392420
Nup214	5’-GGUGAGAAUCUUUGACUCC-3’
Nup93	Horizon ON-TARGETplus siRNA L-020767-01-0005
CLIMP-63	5'-GACAACAUCGCCAUCUUCA-3′
RTN4	Horizon ON-TARGETplus siRNA L-010721-00-0005
p180/RRBP1	Horizon ON-TARGETplus siRNA L-011891-02-0005
KTN1	Horizon ON-TARGETplus siRNA L-010605-00-0005
ATL1	Horizon ON-TARGETplus siRNA L-010946-00-0005
ATL3	Horizon ON-TARGETplus siRNA L-010656-01-0005
LNPK	Horizon ON-TARGETplus siRNA L-023148-01-0005

Guide RNAs	
**Targeted gene**	**Targeted Sequence**
Nup133	CTGTCCTAGTCGCTGCTCCT
UBAP2L-1	TGGCCAGACGGAATCCAATG
UBAP2L-2	GTGGTGGGCCACCAAGACGG
